# Periodic electroencephalographic discharges and epileptic spasms involve cortico-striatal-thalamic loops on Arterial Spin Labeling Magnetic Resonance Imaging

**DOI:** 10.1093/braincomms/fcac250

**Published:** 2022-10-06

**Authors:** Monika Eisermann, Ludovic Fillon, Ana Saitovitch, Jennifer Boisgontier, Alice Vinçon-Leite, Volodia Dangouloff-Ros, Thomas Blauwblomme, Marie Bourgeois, Marie-Thérèse Dangles, Delphine Coste-Zeitoun, Patricia Vignolo-Diard, Mélodie Aubart, Manoelle Kossorotoff, Marie Hully, Emma Losito, Nicole Chemaly, Monica Zilbovicius, Isabelle Desguerre, Rima Nabbout, Nathalie Boddaert, Anna Kaminska

**Affiliations:** Clinical Neurophysiology, Hôpital Necker Enfants Malades, AP-HP, Paris Université, Paris, France; Pediatric Radiology Department, AP-HP, Hôpital Necker Enfants Malades, Université de Paris, F-75015, Paris, France; Université de Paris, Institut Imagine INSERM U1163, F-75015, France; INSERM U1299 Trajectoires développementales & psychiatrie, Paris, France; Pediatric Radiology Department, AP-HP, Hôpital Necker Enfants Malades, Université de Paris, F-75015, Paris, France; Université de Paris, Institut Imagine INSERM U1163, F-75015, France; INSERM U1299 Trajectoires développementales & psychiatrie, Paris, France; Pediatric Radiology Department, AP-HP, Hôpital Necker Enfants Malades, Université de Paris, F-75015, Paris, France; Université de Paris, Institut Imagine INSERM U1163, F-75015, France; INSERM U1299 Trajectoires développementales & psychiatrie, Paris, France; Pediatric Radiology Department, AP-HP, Hôpital Necker Enfants Malades, Université de Paris, F-75015, Paris, France; Université de Paris, Institut Imagine INSERM U1163, F-75015, France; INSERM U1299 Trajectoires développementales & psychiatrie, Paris, France; Pediatric Radiology Department, AP-HP, Hôpital Necker Enfants Malades, Université de Paris, F-75015, Paris, France; Université de Paris, Institut Imagine INSERM U1163, F-75015, France; INSERM U1299 Trajectoires développementales & psychiatrie, Paris, France; Pediatric Neurosurgery, Hôpital Necker, APHP, Paris France, Université de Paris, Paris, France, INSERM U1163, IHU Imagine, Paris, France; Pediatric Neurosurgery, Hôpital Necker, APHP, Paris France, Université de Paris, Paris, France, INSERM U1163, IHU Imagine, Paris, France; Clinical Neurophysiology, Hôpital Necker Enfants Malades, AP-HP, Paris Université, Paris, France; Clinical Neurophysiology, Hôpital Necker Enfants Malades, AP-HP, Paris Université, Paris, France; Clinical Neurophysiology, Hôpital Necker Enfants Malades, AP-HP, Paris Université, Paris, France; Pediatric Neurology Department, Hôpital Necker Enfants Malades, AP-HP, INSERM U1163, Paris Université, Institut Imagine, Paris, France; Pediatric Neurology Department, Necker Enfants Malades Hospital, AP-HP, Paris Université, Paris, France; Pediatric Neurology Department, Necker Enfants Malades Hospital, AP-HP, Paris Université, Paris, France; Clinical Neurophysiology, Hôpital Necker Enfants Malades, AP-HP, Paris Université, Paris, France; Reference Center for Rare Epilepsies, Department of Pediatric Neurology, Member of EPICARE Network, Institute Imagine INSERM 1163, Université de Paris, Paris, France; Pediatric Radiology Department, AP-HP, Hôpital Necker Enfants Malades, Université de Paris, F-75015, Paris, France; Université de Paris, Institut Imagine INSERM U1163, F-75015, France; INSERM U1299 Trajectoires développementales & psychiatrie, Paris, France; Pediatric Neurology Department, Hôpital Necker Enfants Malades, AP-HP, INSERM U1163, Paris Université, Institut Imagine, Paris, France; Reference Center for Rare Epilepsies, Department of Pediatric Neurology, Member of EPICARE Network, Institute Imagine INSERM 1163, Université de Paris, Paris, France; Pediatric Radiology Department, AP-HP, Hôpital Necker Enfants Malades, Université de Paris, F-75015, Paris, France; Université de Paris, Institut Imagine INSERM U1163, F-75015, France; INSERM U1299 Trajectoires développementales & psychiatrie, Paris, France; Clinical Neurophysiology, Hôpital Necker Enfants Malades, AP-HP, Paris Université, Paris, France

**Keywords:** ASL-MRI, periodic EEG discharges, epileptic spasms, basal ganglia, thalamus

## Abstract

Periodic discharges are a rare peculiar electroencephalogram pattern, occasionally associated with motor or other clinical manifestations, usually observed in critically ill patients. Their underlying pathophysiology remains poorly understood. Epileptic spasms in clusters and periodic discharges with motor manifestations share similar electroencephalogram pattern and some aetiologies of unfavourable prognosis such as subacute sclerosing panencephalitis or herpes encephalitis. Arterial spin labelling magnetic resonance imaging identifies localizing ictal and inter-ictal changes in neurovascular coupling, therefore assumed able to reveal concerned cerebral structures. Here, we retrospectively analysed ictal and inter-ictal arterial spin labelling magnetic resonance imaging in patients aged 6 months to 15 years (median 3 years 4 months) with periodic discharges including epileptic spasms, and compared these findings with those of patients with drug-resistant focal epilepsy who never presented periodic discharges nor epileptic spasms as well as to those of age-matched healthy controls. Ictal electroencephalogram was recorded either simultaneously with arterial spin labelling magnetic resonance imaging or during the close time lapse of patients’ periodic discharges, whereas inter-ictal examinations were performed during the patients’ active epilepsy but without seizures during the arterial spin labelling magnetic resonance imaging. Ictal arterial spin labelling magnetic resonance imaging was acquired in five patients with periodic discharges [subacute sclerosing panencephalitis (1), stroke-like events (3), West syndrome with cortical malformation (1), two of them also had inter-ictal arterial spin labelling magnetic resonance imaging]. Inter-ictal group included patients with drug-resistant epileptic spasms of various aetiologies (14) and structural drug-resistant focal epilepsy (8). Cortex, striatum and thalamus were segmented and divided in six functional subregions: prefrontal, motor (rostral, caudal), parietal, occipital and temporal. Rest cerebral blood flow values, absolute and relative to whole brain, were compared with those of age-matched controls for each subregion. Main findings were diffuse striatal as well as cortical motor cerebral blood flow increase during ictal examinations in generalized periodic discharges with motor manifestations (subacute sclerosing panencephalitis) and focal cerebral blood flow increase in corresponding cortical-striatal-thalamic subdivisions in lateralized periodic discharges with or without motor manifestations (stroke-like events and asymmetrical epileptic spasms) with straight topographical correlation with the electroencephalogram focus. For inter-ictal examinations, patients with epileptic spasms disclosed cerebral blood flow changes in corresponding cortical-striatal-thalamic subdivisions (absolute-cerebral blood flow decrease and relative-cerebral blood flow increase), more frequently when compared with the group of drug-resistant focal epilepsies, and not related to Vigabatrin treatment. Our results suggest that corresponding cortical-striatal-thalamic circuits are involved in periodic discharges with and without motor manifestations, including epileptic spasms, opening new insights in their pathophysiology and new therapeutical perspectives. Based on these findings, we propose a model for the generation of periodic discharges and of epileptic spasms combining existing pathophysiological models of cortical-striatal-thalamic network dynamics.

## Teaser

Eisermann *et al*. report from arterial spin labelling magnetic resonance imaging studies in children that cortico-striatal-thalamic circuits are involved in periodic electroencephalogram discharges and epileptic spasms and propose a model for their generation by combining existing pathophysiological models of cortico-striatal-thalamic network dynamics.

## Graphical Abstract

**Graphical abstract:**
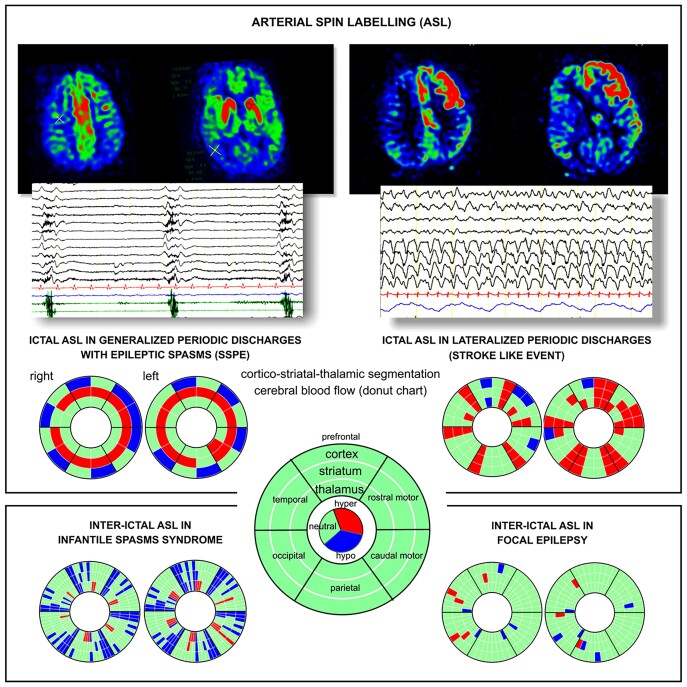

## Introduction

Periodic discharges (PDs) on electroencephalogram (EEG) have been reported in adults and children in numerous acute or chronic central nervous system (CNS) diseases, generally considered as of unfavourable prognosis.^1–3^ PDs are defined as a ‘repetition of a waveform with relatively uniform morphology and duration, with a definable interval between consecutive waveforms and recurrence of the waveform at nearly regular intervals’.^4^ They are categorized regarding their waveform, phases, topographic distribution [generalized PDs (GPDs), lateralized PDs (LPDs)] and periodicity.^4–7^ When PDs are time locked to clinically apparent signs, i.e. motor focal (epilepsia partialis continua) or diffuse myoclonic jerks or spasms, oculomotor (eyeball jerking), visual manifestations or visual or auditory hallucinations, they are usually considered ictal, regardless of their frequency.^1^ Interestingly, epileptic spasms (ESs) occurring in clusters share some of these electrophysiological criteria with PDs with motor manifestations. ES can be focal or generalized, are concomitant to a lateralized or diffuse slow wave or spike wave on EEG, occur in a periodic manner, however with variable periodicity and morphology of the EEG pattern inside the cluster, and occur mainly during the transition between vigilance states.^8–10^ In subacute sclerosing panencephalitis (SSPE), PDs with concomitant jerks accomplish the criteria of ES since the muscular contraction may be diamond shaped and not shock like as in myoclonus and is concomitant to a diffuse slow-wave EEG complex.^1,11^ It is noteworthy that, like for aetiologies related to GPDs, ESs occur mostly in patients with severe CNS diseases and of multiple aetiologies, also in large life span from neonatal period to adulthood.^11–16^ GPDs with jerks in SSPE are therefore an interesting model to study the pathophysiological mechanism of generalized ES and of PDs with motor features.

Arterial spin labelling magnetic resonance imaging (ASL-MRI) is a non-invasive technique which quantifies tissue blood flow using magnetically labelled blood as an endogenous contrast agent. ASL-MRI is a well-established method for task-specific neuronal activation imaging^17,18^ and has been shown to identify localizing changes in neurovascular coupling in focal epilepsy in children^19,20^ as well as in patients with PDs.^21,22^ Thus, ASL-MRI imaging may be suitable to reveal the underlying cerebral structures involved in PD generation.

Epileptic spasms in clusters and PDs share many electro-clinical characteristics as aetiologies (e.g. SSPE, herpes encephalitis), similarity of EEG pattern, periodicity and ictal phenomenology,^11^ but their pathophysiological mechanism remains poorly understood. In order to disclose their cerebral generators, we retrospectively analysed ictal cerebral blood flow (CBF) changes in patients having presented PDs (GPDs or LPDs) or ES during ASL-MRI acquisition. Moreover, we assessed inter-ictal CBF changes (neither ES nor PDs during ASL-MRI) in patients with daily ES, and compared these findings to those of inter-ictal ASL-MRI (no focal seizures during ASL-MRI) of patients with drug-resistant focal epilepsy (DRFE) and daily seizures but without history of PDs or ES. As previous functional imaging studies including ASL-MRI showed cortical and subcortical structures involved in patients with PDs as well as in ES,^21–24^ ASL-MRI was analysed in striatal (S), thalamic (T) and cortical (C) regions based on atlases allowing functional subdivision of these regions.^25,26^

## Materials and methods

**Figure 1:**
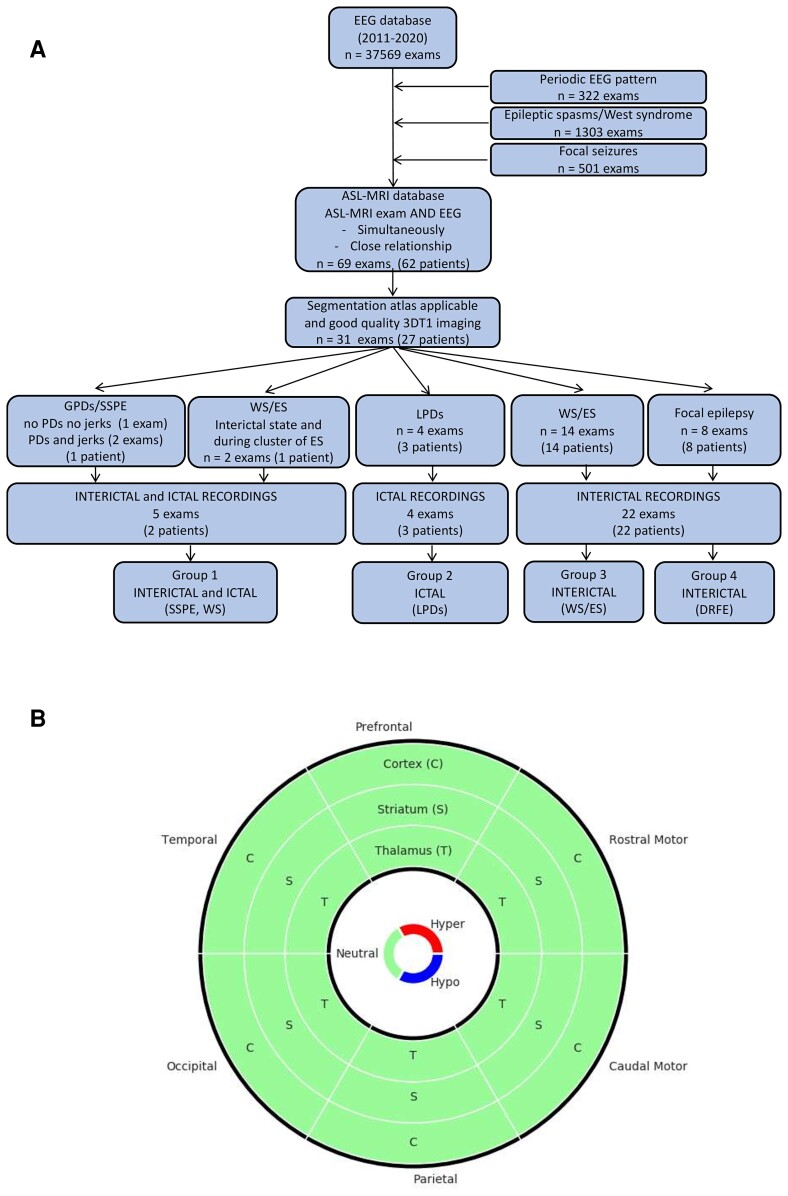
**Flow chart and donut chart.** (**A**) Flow chart. Inclusion of patients and examinations. Patients and examinations were identified by screening the EEG database between January 2011 (beginning of systematic ASL-MRI recordings in our hospital) and November 2020 for patients presenting with PDs, patients with daily ES and patients with DRFE with daily seizures without history of PDs or ES. In order to assess ictal and/or inter-ictal ASL-MRI changes in these patients, we retrospectively included those who had undergone an ASL-MRI study in close temporal relationship to the video EEG, or recorded simultaneously (EEG-ASL-MRI—part of an ongoing prospective study). In patients presenting with PDs, we assumed that PDs were present during the ASL-MRI study by the fact that video EEG before and after ASL-MRI (maximum interval 7 days) were comparable, and the patients did not recover or receive additional ASM able to stop PDs. In patients with ES and DRFE with daily seizures, we included those who underwent video EEG within 1 month before/after ASL-MRI to ensure that their EEG features were still compatible with the diagnosis of their epilepsy type and syndrome. During the ASL-MRI, the status was considered as inter-ictal when no seizure was observed nor recorded (if simultaneous EEG). (**B**) Donut chart used to represent cortico-striatal-thalamic circuits. This chart is composed of three rings for cortex (C), striatum (S) and thalamus (T), respectively, from outside to inside. The chart is divided into six equal parts corresponding to the six subdivisions studied, clockwise: prefrontal, rostral motor, caudal motor, parietal, occipital and temporal. For each compartment, if the two-sample *t*-test of the studying ROI is significant (*P* < 0.05), the colour is red if the patient’s A-CBF (or R-CBF) value is greater than the average of the controls’ A-CBF (or R-CBF) values, blue if the patient’s A-CBF (or R-CBF) value is lower than the average of the controls’ A-CBF (or R-CBF) values, and green if the test is not significant.

Patients were followed at the reference centre for rare epilepsies, at Necker Enfants Malades University Hospital, Paris, France. The study was approved by the institutional ethics committee (CENEM2019-17-MCV) and was listed in the general registry of data treatment (20190725161307). Figure 1A (flow chart) details identification and inclusion of patients and examinations. Twenty-seven patients were included (11 males) and classified into four groups according to ictal or inter-ictal state during the ASL-MRI and to their epilepsy type or syndrome (epilepsy with ES or DRFE). Electro-clinical features are detailed in Supplementary Table 1.

**Group 1:** Two patients with ASL-MRI acquired during both inter-ictal and ictal states. Patient 1: Fifteen years old previously healthy boy, presented at age 14 years with frontal seizures followed by fast psychomotor regression progressing within 4 m to severe encephalopathy with minimal responsiveness, spastic tetraparesis and jerks of the four limbs. EEG disclosed concomitant GPDs leading to the diagnosis of SSPE confirmed by the presence of measles antibodies in cerebrospinal fluid. Patient 26: Six months old boy started asymmetrical ES (predominating on the right arm) at age 4.5 months. EEG showed hypsarrhythmia in wake and sleep, brain MRI disclosed left extended posterior predominant and right temporal cortical malformation.

**Group 2 (ictal):** Three patients (2–4; two males), with stroke-like events presenting PDs during ASL-MRI. Two patients had two stroke-like events, thus a total of five ASL-MRI were performed at the age of 5 and 6 months (Patient 2), 12 years (Patient 3), 11 years 11 months and 13 years 3 months (Patient 4). Patient 2 presented a pathological variant in the *SCN1A* gene and siblings (Patients 3 and 4) an undetermined aetiology (metabolic testing and whole-genome sequencing negative).

**Group 3 (inter-ictal):** Fourteen patients with ES (5–16, 24, 25; 5 males), aged 6 months to 6 years 10 months (median 8 months) at the time of the ASL-MRI. All had drug-resistant ES [12 West syndrome (WS) and 2 late-onset ES] occurring at least once a day, isolated or in clusters. Aetiologies were multiple, post-infectious (following herpes encephalitis), auto-immune encephalitis, chromosomal rearrangements, structural cortical anomalies, sequelae of prematurity, unknown in four patients. All but one patient (24) showed severe background EEG anomalies.

**Group 4 (inter-ictal):** Eight patients with DRFE (17–23, 27; two males), without history of PDs nor ES, aged 6 months to 15 years 7 months (median 9 years 11 months) at ASL-MRI. Median duration of epilepsy was 4 years 7 months, the underlying aetiology in all patients structural.

A control group composed of 232 children was selected to match for age, MRI scanner and sedation status of each patient. Controls underwent ASL-MRI for various conditions presenting strictly normal scans, thus considered not affecting the cerebrum (Supplementary Material Methods, Supplementary Fig. 1A–C).

### Electroencephalography

Video EEGs were recorded on Deltamed/Coherence EEG system (Natus, France) lasting 1–24 h, including polygraphic parameters [electrocardiogram, respiration, surface electromyography (EMG) on both deltoid muscles, using 21 silver chloride cup electrodes placed according to the 10/20 international system with medial frontal polar as reference electrode (for details see Supplementary Material Methods).

### MRI and ASL-MRI acquisition

For both patients and controls, all images were acquired with a standard routine protocol including whole-brain T_1_-weighted and ASL sequences, detailed in Supplementary Material Methods. For each patient, type and topography of the cerebral lesion, if present, were assessed (Supplementary Table 1).

### Electroencephalogram—arterial spin labelling magnetic resonance imaging

For EEGs realized in seven patients (nine examinations) concomitantly to MRI in an ongoing prospective study, an MRI-compatible EEG cap with 31 electrodes (Micromed, Italy), an MRI-compatible amplifier (Micromed, Italy) and a device synchronizing EEG to the MRI scanner clock were used. Gradient and pulse artefacts were corrected using the integrated Micromed Software. Prior to scanning, EEG was recorded outside the scanner for 15 min under standard conditions.

### ASL-MRI data analysis

We used quantified CBF images (ml/100 g/min) for data preprocessing and statistical analysis. CBF images were automatically generated and warranted with General Electric’s 3D ASL processing console, requiring a proton-density image for signal normalization during CBF quantification (see Appendix E1 in Zaharchuk *et al*.^27^).

All 3D T_1_-weighted and ASL images were first analysed by visual inspection for main cortical and subcortical CBF changes and checked for major artefacts, such as motion, aliasing, ghosting, spikes, low signal to noise ratio, by an expert radiologist right after acquisition (N.B.) and by an expert image processing engineer (L.F.) before preprocessing. All images with artefacts were discarded. All MR images were preprocessed using Statistical Parametric Mapping 12 (SPM12; http://www.fil.ion.ucl.ac.uk/spm) within MatlabR2018b. Native 3D T_1_-weighted images were preprocessed using the Computational Anatomy Toolbox (CAT; http://www.neuro.uni-jena.de/cat/) for SPM12 and segmented into grey matter, white matter and cerebrospinal fluid using a Montreal Neurological Institute (MNI) template adapted to the age of the studied child. For children under 1 year old, we used the Infant Brain Probability Templates.^28^ For children over 1 year old, we used a paediatric template created with ‘Template-O-Matic’ toolbox (http://dbm.neuro.uni-jena.de/software/tom/) using the NIH Pediatric MRI Data Repository (*n* = 404, age range 5–18 years). The co-registration was estimated between the native CBF images and the corresponding native grey-matter images. Then, CBF images were spatially normalized in the MNI space applying the combination of the estimated co-registration and the deformation fields from the CAT12 segmentation. Substriatal and subthalamic regions of interest (ROI) were defined using the fsl-oxford-striatal-atlas^25^ and fsl-oxford-thalamic-connectivity-atlas.^26^ In these atlases, S and T were divided into seven subregions segmented according to their white matter connectivity to cortical areas using probabilistic diffusion tractography. Substriatal regions were defined as limbic, executive, rostral motor (RM) (precentral, supplementary motor area [SMA]), caudal motor (CM) (prerolandic/primary motor), parietal, occipital and temporal. Subthalamic regions were defined as prefrontal, premotor (precentral, SMA), primary motor, sensory, posterior parietal, occipital and temporal. To match the subdivisions of the two atlases, the limbic and executive ROI of S atlas were merged to delineate the prefrontal ROI, and for T, the sensory and posterior parietal ROI were merged to delineate the parietal ROI. Thus, a total of six subdivisions were defined for S and T: prefrontal, RM (precentral, SMA), CM (prerolandic, primary motor), parietal, occipital and temporal. In order to have a cortical-striatal-thalamic association, corresponding cortical ROIs were defined using the automated anatomical labelling atlas.^29,30^ For each hemisphere, mean CBF values in each ROI of C, S and T were obtained from normalized CBF images (absolute CBF: A-CBF). In addition, we scaled each ROI mean CBF value by the mean CBF value of the whole-brain cortex to obtain ratio values [ratio CBF (R-CBF)].

### Statistical analysis

Independent two-sample *t*-tests based on A-CBF and R-CBF were computed per hemisphere for each ROI of C, S and T between each patient and the age- and scanner-matched control group (Supplementary Material Methods; Supplementary Fig. 1). We assumed that the A-CBF values would reveal a localized increase or decrease in CBF in patients when compared with the norm given by controls. In addition, we assumed that R-CBF values would reveal a relative intraindividual increase or decrease in CBF in potential subregions in patients. Compared with controls, these R-CBF values would indicate whether this ratio was increased, normal or decreased. Furthermore, Fisher's exact test was applied for qualitative variables. All statistical analyses were performed using Python 3.7.3 and scipy.stats module v1.2.1. Only *P*-values under 0.05 were considered significant.

### Data availability statement

The data sets generated during and/or analysed during the current study are available within the article and its supplementary material. Additional anonymized data are available from the corresponding author on reasonable request.

## Results

### Group 1—ASL-MRI-EEG in both inter-ictal and ictal states

**Figure 2:**
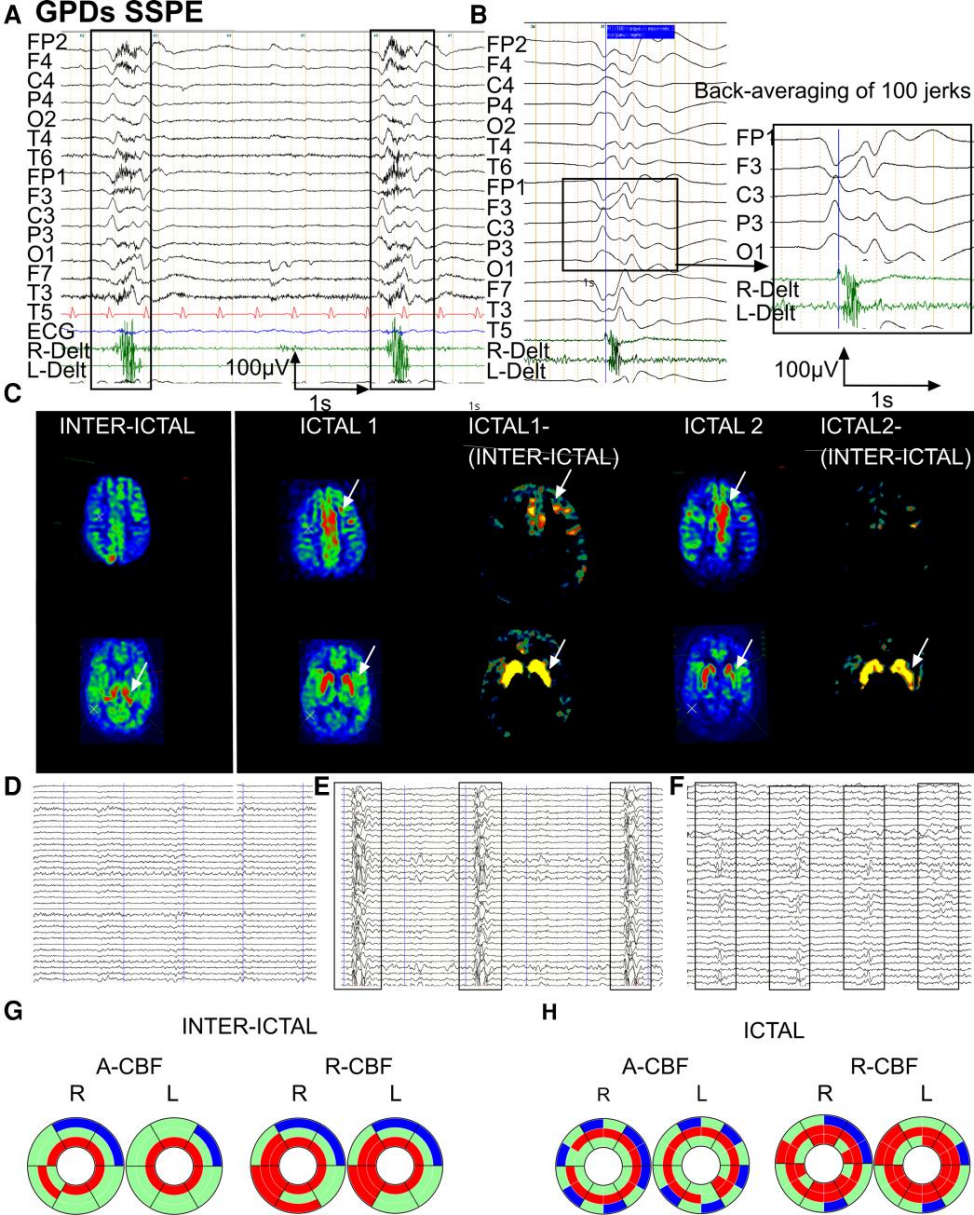
**Inter-ictal and ictal EEG-ASL-MRI in Patient 1, 15 years old, SSPE.** (**A**) EEG with surface EMG polygraphic recording. Common average reference montage. Periodic diffuse high-amplitude slow-wave complexes of stereotyped morphology with maximum amplitude on left central-parietal leads occurring on a depressed background activity. Surface EMGs (deltoid muscles) of the concomitant jerks show a bilateral contraction beginning and predominating on the right deltoid. (**B**) Jerk-locked back-averaging (100 jerks), common average reference montage. Bilateral negative central-parietal sharp wave predominating on the left, reaching a maximal amplitude of 90µV with phase reversal over frontal areas. (**C**) ASL-MRI imaging in inter-ictal and ictal state [two examinations: ictal1 (1b) and ictal2 (1c) at 7 months interval] with ictal-(inter-ictal) subtraction. Arrows indicate structures with significant CBF increase. Compared with the inter-ictal state, ictal CBF was increased in frontal mesial and left frontal cortex. CBF in thalamus was increased in the inter-ictal state, whereas CBF here was normal in both adjacent cortex and in striatum. In ictal1 and ictal2 examinations, striatum showed increased CBF, while here CBF was unaffected in thalamus. (**D**) Simultaneous EEG recording during the ASL-MRI examination (1a). Slow and depressed background activity, neither GPDs nor jerks present. (**E**) Simultaneous EEG recording during the ASL-MRI examination (1b). GPDs concomitant with jerks on depressed background activity with complex-to-complex intervals of 5–7 s. (**F**) Simultaneous EEG recording during the ASL-MRI examination (1c). GPDs concomitant with jerks on depressed background activity showing shorter complex-to-complex intervals with further clinical evolution, of 2–3 s. (**G**) Donut charts (for details see Fig. 1B). Significant A-CBF and R-CBF changes compared with controls during inter-ictal state. A-CBF increased in all but one thalamic subdivisions and the right occipital striatum. R-CBF increased in all thalamic compartments as well as in bilateral temporal striatum, and in bilateral occipital C-S-T circuits. (**H**) Donut charts (for details see Fig. 1B and Supplementary Fig. 2). Significant A-CBF and R-CBF changes compared with controls during inter-ictal and both ictal examinations. A-CBF increased in all but two striatal subdivisions and in left caudal motor thalamus. R-CBF increased in all striatal and in most thalamic subdivisions, as well as in temporal cortical compartments.

**Patient with SSPE:** PDs occurred on a depressed background activity consisting in diffuse, initially high, later in the evolution lower amplitude polyphasic slow-wave complexes of stereotyped morphology (Fig. 2A). On video EEG, GPDs were concomitant with jerks affecting the four limbs and the head, associated with a perioral contraction and a single brief eye-opening movement. Deltoid EMG showed bilateral contraction beginning and predominating on the right side, in awake state (Fig. 2A), diminishing in intensity or disappearing in sleep. The concomitant motor phenomenon was characterized on surface EMG by a brief diamond-shaped contraction of variable duration (Fig. 2A). Jerk-locked back-averaging showed a bilateral left predominant negative central-parietal sharp wave, with phase reversal over frontal areas preceding the right deltoid EMG contraction with a variable latency ranging from 20 to 60 ms (Fig. 2B). He underwent three EEG-ASL-MRI: the first (1a) was realized during a period where neither jerks nor periodic EEG complexes were present (Fig. 2C and D); during the second and third (1b and 1c), periodic jerks were present and time locked to the GPDs (Fig. 2C, E, F).

When compared with the inter-ictal state (visual inspection and subtraction), ictal ASL-MRI disclosed CBF increase in bilateral central and frontal mesial cortex as well as in bilateral striatum (Fig. 2C); whereas on inter-ictal ASL-MRI, mainly thalamus disclosed increased CBF bilaterally (Fig. 2C). When compared with controls, inter-ictal ASL-MRI showed significant A-CBF increase in all but one thalamic subdivisions and the right occipital striatal one (Fig. 2G). R-CBF values during the inter-ictal examination were increased in all thalamic compartments and in bilateral temporal striatal, as well as in bilateral occipital cortico-striatal-thalamic circuits (Fig. 2G). Both ictal ASL-MRI showed A-CBF increase in all but two striatal subdivisions and in left CM thalamic one (Fig. 2H). The ictal cortical CBF increase in central and frontal regions seen on visual inspection however did not reach statistical significance when compared with controls. Ictal R-CBF was increased during both examinations in all striatal and in most thalamic subdivisions, as well as in temporal cortex (Fig. 2H; Supplementary Figs. 1B, C and 2).

**Figure 3:**
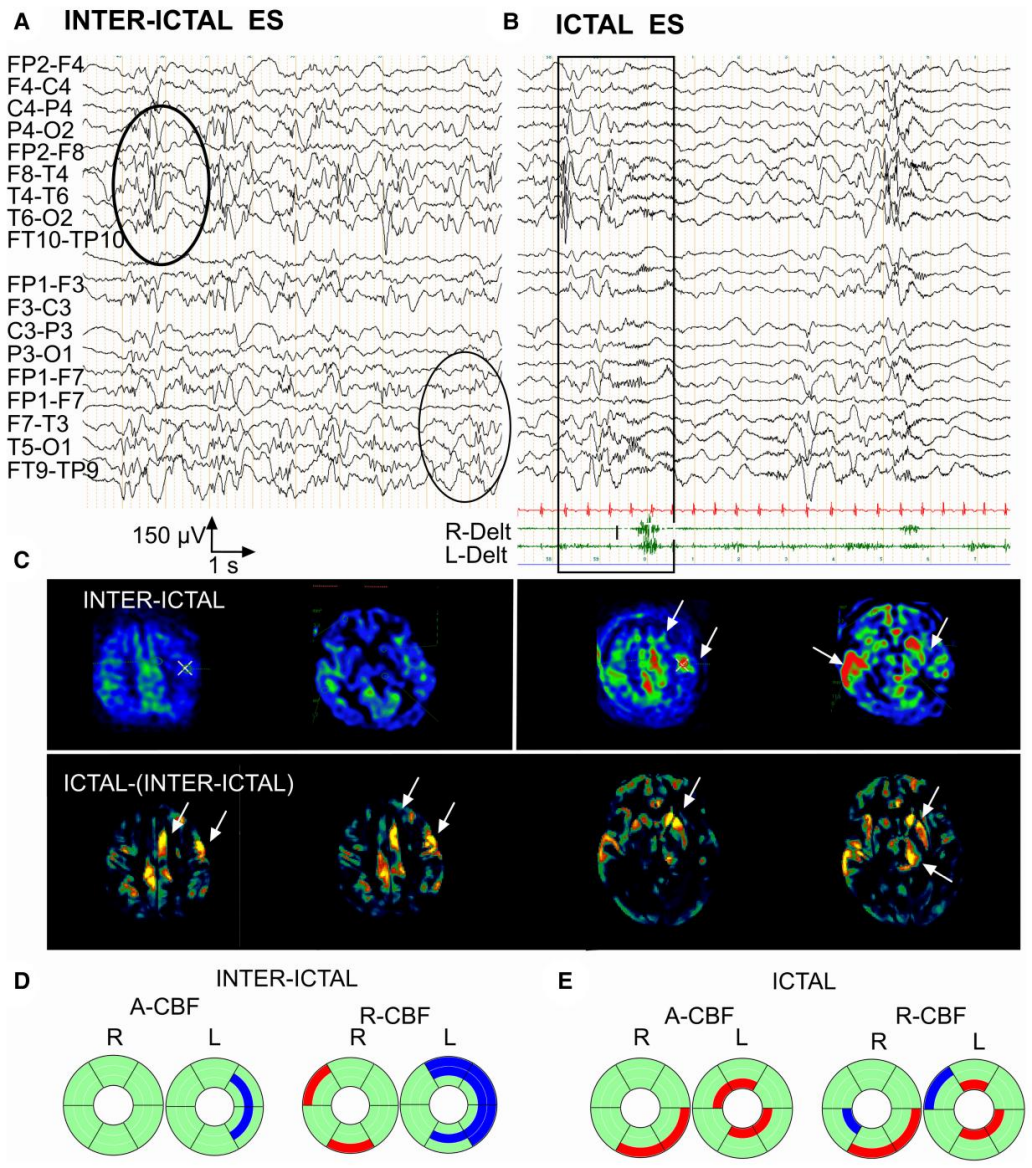
**Inter-ictal and ictal EEG-ASL-MRI in Patient 26, 6 months old, epileptic spasms/West syndrome with bilateral extended cortical dysplasia.** (**A**) Inter-ictal EEG, awake. Independent slow-wave and spike-wave foci in left and right central-parietal-temporal regions. (**B**) Ictal EEG with surface EMG polygraphic recording, bipolar montage. Asymmetric epileptic spasm predominating on the right deltoid muscle concomitant on EEG with a diffuse slow-wave complex associated with low-voltage, diffuse fast rhythm bursts predominating on left parietal-temporal region. (**C**) ASL-MRI imaging in inter-ictal and ictal state with ictal-(inter-ictal) subtraction. Arrows indicate structures with significant CBF increase. Ictal CBF is increased in central-mesial and left frontal cortex compared with their inter-ictal CBF. Ictal CBF was also increased in the left striatum, left thalamus and right temporal cortex compared with their inter-ictal CBF. (**D**) Donut charts (for details, see Fig. 1B and Supplementary Fig. 3). Significant A-CBF and R-CBF changes compared with controls during inter-ictal state. A-CBF decreased in two right S (rostral motor, caudal motor). R-CBF increased in two right C (parietal, temporal) and decreased in three corresponding left C and S (prefrontal, rostral motor, caudal motor) and in left parietal S. (**E**) Donut charts (for details, see Fig. 1B and Supplementary Fig. 3). Significant A-CBF and R-CBF changes compared with controls during ictal state. A-CBF increased in four left T (prefrontal, caudal motor, parietal and temporal) and in two right C (caudal motor, parietal). R-CBF increased in three left T (prefrontal, caudal motor, parietal) and two right-sided C (parietal, caudal motor).

**Patient with ES/West syndrome:** Inter-ictal EEG showed two independent slow-wave and spike-wave foci in left and right central-parietal-temporal regions (Fig. 3A). EEG-ASL-MRI was recorded in inter-ictal state (26a) and during a cluster of asymmetric, clinically right-sided ES (26b) with on EEG a diffuse slow-wave complex associated with low-voltage diffuse fast rhythm bursts predominating on left parietal-temporal region (Fig. 3B).

When compared with the inter-ictal state (visual inspection, subtraction), ictal ASL-MRI showed CBF increase in bilateral but left predominant striatum, thalamus and motor and frontal mesial cortex as well as in the right central-parietal-temporal cortex (Fig. 3C). When compared with controls, A-CBF during ictal examination was significantly increased in left thalamic compartments (prefrontal, CM, parietal, and temporal) and in the right CM and parietal cortex (Fig. 3E; Supplementary Fig. 3). Ictal R-CBF, when compared with controls, was increased in three left-sided thalamic subdivisions (prefrontal, CM, and parietal) and two right-sided cortical (parietal and CM; Fig. 3E; Supplementary Figs. 1B, C and 3).

### Group 2 (ictal)—PDs

Motor manifestations accompanied PDs in three of the four ASL-MRI examinations (2, 3 and 4b), and in one examination (4a), PDs were subclinical.

**Figure 4:**
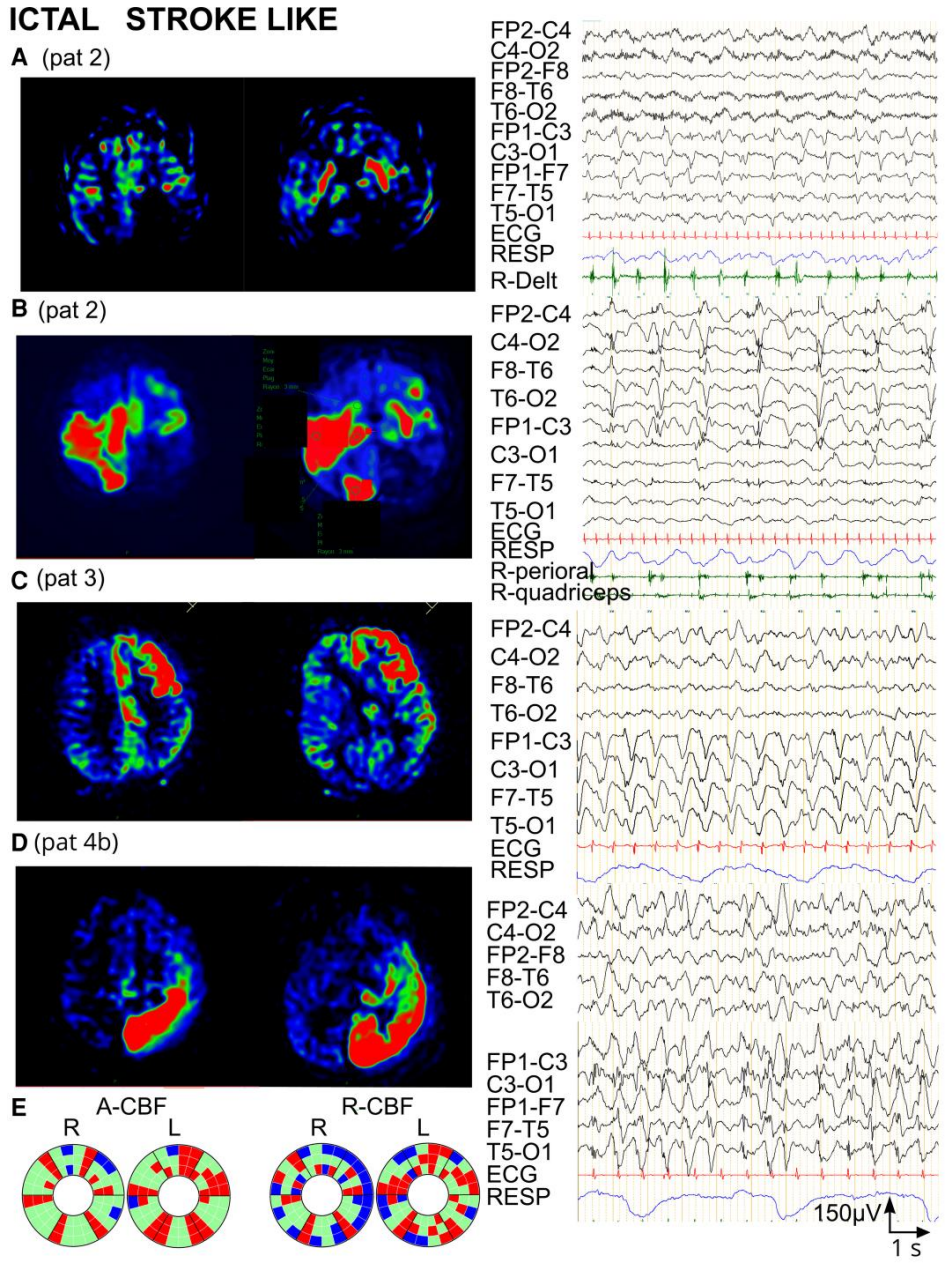
**Ictal ASL-MRI in patients with periodic discharges during stroke-like events (Group 2).** (**A**) Patient 2, 5 months old, pathological variant in *SCN1A*. ASL-MRI during first stroke-like event (segmentation atlas not applicable, no statistical comparison with controls possible). Left: ASL-MRI. Bilateral S and left central CBF increase. Right: EEG with right deltoid EMG recording. Left frontal-central periodic complexes concomitant with right upper limb clonic jerks. (**B**) Same patient as in **A**, 6 months old. ASL-MRI during second stroke-like event. Left: ASL-MRI. Bilateral CBF increase (right central-temporal-occipital and left frontal-central). Right: EEG with right perioral and quadriceps EMG. Right central-mesial and temporal-occipital PDs, and left frontal-central PDs associated clinically with right-sided lower limb and right perioral jerks. (**C**) Patient 3, 12 years old, stroke-like event of unknown aetiology. Left: ASL-MRI. A-CBF increase in left-sided prefrontal C-S-T and rostral motor S-T. Right: EEG. High voltage left hemispheric PDs with frontal-central predominance, associated clinically with eyelid jerks. (**D**) Patient 4 (examination 4b), 13 years old, stroke-like event of unknown aetiology. Left: ASL-MRI. A-CBF increase in left parietal, occipital and temporal C-S-T and in left prefrontal, rostral and caudal motor T. Right: EEG. Left hemispheric PDs accompanied by right lower limb jerks. (**E**) Donut charts (for details, see Fig. 1B and Supplementary Fig. 4) including the four ASL-MRI examinations in the three patients. Left: significant A-CBF changes compared with controls, observed in 40% of all compartments, most frequently increased and concerning corresponding C-S-T. Right: significant R-CBF changes compared with controls, observed in 53% of all compartments, frequently decreased, especially in C.

Patient 2 presented left-sided stroke-like event with left frontal-central PDs concomitant to right upper limb clonic jerks (Fig. 4A). ASL-MRI showed bilateral striatal and left central cortical CBF increase (segmentation non feasible, therefore not included into statistical CBF analysis/controls; Fig. 4A). At age 6 months, she presented a second, bilateral stroke-like event with right central-mesial and temporal-occipital PDs as well as left frontal-central PDs associated with right facial and lower limb jerks (Fig. 4B). A-CBF was increased in corresponding C-S-T circuits in both hemispheres: left prefrontal, RM, CM, parietal, and right prefrontal, CM, parietal, temporal and occipital (Fig. 4E, Supplementary Fig. 4).

The EEG of Patient 3 showed at the time of the left frontal stroke-like event left hemispheric PDs with frontal-central predominance associated with eyelid jerks (Fig. 4C). A-CBF was increased in left-sided prefrontal C-S-T and RM S-T circuits (Fig. 4E; Supplementary Fig. 4).

In Patient 4, EEG at first ASL-MRI showed bilateral independent right predominant frontal-central PDs without concomitant motor manifestations. A-CBF was increased in right RM and temporal thalamic subdivisions, and in left RM striatum. R-CBF was increased in left temporal S-T circuit and four other left striatal compartments (prefrontal, RM, CM and parietal; Fig. 4E; Supplementary Fig. 4). At second stroke-like event in Patient 4, EEG disclosed left hemispheric PDs accompanied by right lower limb jerks (Fig. 4D). A-CBF was increased in left prefrontal, RM and CM thalamus and in left parietal, occipital and temporal corresponding C-S-T circuits (Fig. 4E; Supplementary Fig. 4).

Overall, among all compartments in this group (144), A-CBF changes were observed in 58 (40%), most frequently increased (51/58) and concerning corresponding C-S-T circuits. Moreover, in all patients, the topography of LPDs corresponded to the localization of A-CBF increase (Fig. 4E; Supplementary Table 1; Supplementary Fig. 4). Regarding R-CBF analysis, when compared with A-CBF, more C-S-T compartments (53%) disclosed perfusion changes, however with higher variability of C-S-T combinations and more frequent R-CBF decrease, especially in cortex (Fig. 4E; Supplementary Figs. 1B, C and 4).

### Group 3 (inter-ictal)—ES

**Figure 5:**
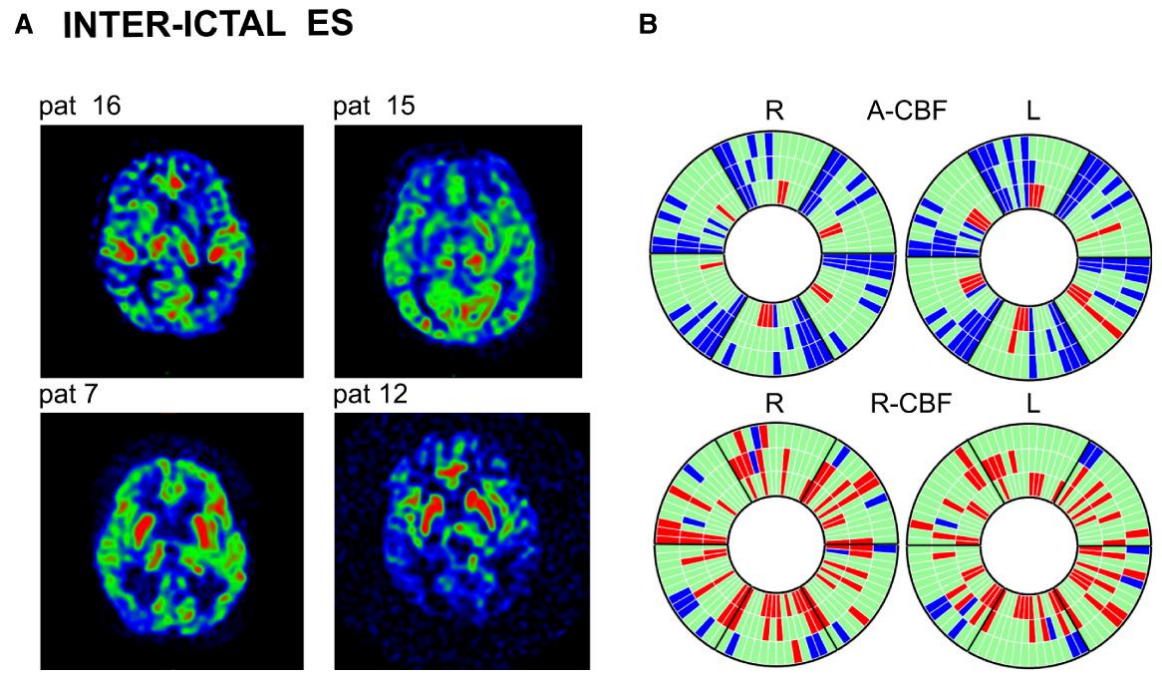
**Inter-ictal ASL-MRI in patients with epileptic spasms (Group 3).** (**A**) ASL-MRI in four representative patients with epileptic spasms (Patients 16, 15, 7 and 12). Patients 15 and 16: examples for A-CBF increase in thalamus and A-CBF decrease in cortex. Patients 7 and 12: examples for R-CBF increase in striatum and the R-CBF decrease in cortex. (**B**) Donut charts (for details, see Fig. 1B and Supplementary Fig. 5) including ASL-MRI examinations of all 14 patients showing significant A-CBF and R-CBF changes compared with controls in this group. A-CBF decrease in corresponding C-S-T, C-S, C-T or S-T compartments. A-CBF increase in T in four patients (10, 14, 15 and 16), all aged <1 year, in one of them (15) associated to homologous S or S-C A-CBF increase.

Seven of 14 patients showed A-CBF changes in corresponding C-S-T, C-S, C-T or S-T circuits, mostly decrease (5/7). A-CBF decrease was more frequent in children >1 year (*P* = 0.023). Thalamic A-CBF increase was observed in four patients, all aged <1 year (*P* = 0.22), in one of them associated to homologous striatal or striatal and cortical A-CBF increase (Fig. 5A; Supplementary Fig. 5). R-CBF increase in corresponding C-S-T, S-T or C-S circuits was observed in 10/14 patients. One patient presented neither R-CBF nor A-CBF changes (Fig. 5B; Supplementary Fig. 5).

Overall, A-CBF changes in this group were observed in 34% of all compartments (170/504), mainly decreased (140/170), with decrease concerning 17% of homologous C-S-T compartments. A-CBF increase was observed in 6% of all compartments, mostly in thalamus. Regarding R-CBF, changes were observed in 32% of all compartments, mainly superior to controls, concerning 21% of corresponding S-T circuits, 16% of striatal and 7% of thalamic compartments (Fig. 5B, Supplementary Fig. 5).

Regarding the antiseizure medication (ASM) at the time of the ASL-MRI possibly impacting CBF, four patients received oral steroids and nine Vigabatrin (VGB). Three of the five patients with A-CBF decrease in C-S-T, C-S, C-T or S-T circuits were on steroids; however, the relationship did not reach significance (*P* = 0.095). The relationship between thalamic and/or striatal A-CBF increase and VGB medication was not significant, neither for the patients <1 year of age (*P* = 0.44) nor for the whole ES group (*P* = 0.22). As for A-CBF, R-CBF increase in striatal and/or thalamic compartments was not correlated with VGB medication (*P* = 0.51).

### Group 4 (inter-ictal)—DRFE

**Figure 6:**
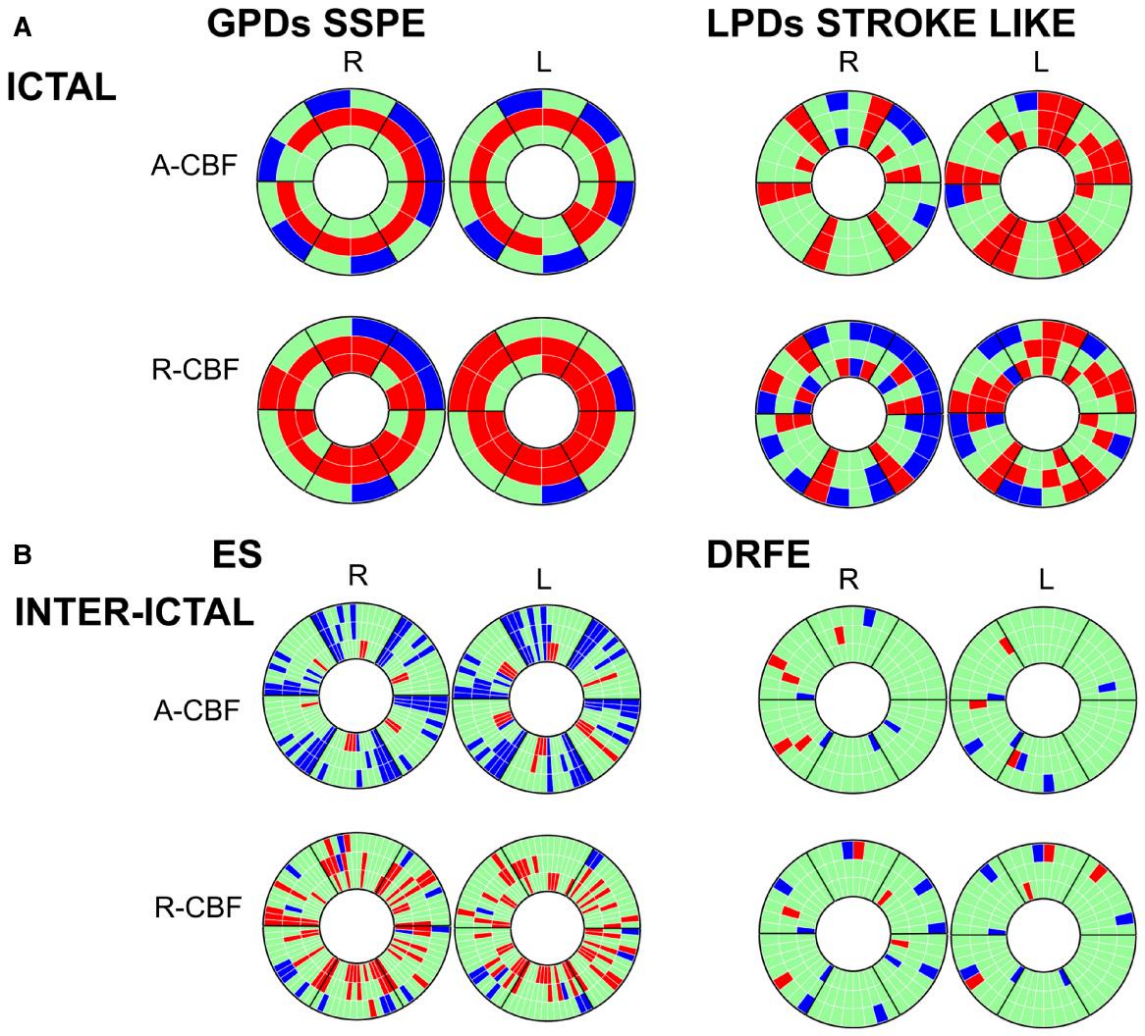
**Overview on donut charts in the four groups (for details, see Fig. 1B) showing significant A-CBF and R-CBF changes compared with controls.** Red: increase, blue: decrease. (**A**) Ictal: GPDs/SSPE (left), LPDs stroke-like (right). See also Supplementary Figs. 2 and 4. (**B**) Inter-ictal: Epileptic spasms (left), focal epilepsy (right). See also Supplementary Figs. 5 and 6.

No CBF changes were observed in corresponding cortical, striatal or thalamic circuits. Isolated CBF changes in striatum, thalamus or cortex were present in five patients, concerning only 7% of all compartments (19/288), with decrease (11/19) or increase (8/19), similar to R-CBF changes (Fig. 6B; Supplementary Fig. 6).

When comparing ES and DRFE groups, we found more frequent A-CBF changes in the ES group, mainly decrease in corresponding C-S-T, C-S, C-T or S-T circuits (*P* = 0.022). R-CBF increase of corresponding S-T circuits was more frequent in ES group (*P* = 0.022) as well as striatal or thalamic R-CBF increase (*P* = 0.024 and *P* = 0.006, respectively; Fig. 6, Supplementary Fig. 1B and C).

## Discussion

In this study, we aimed to investigate pathophysiological mechanisms underlying PDs. Our main findings were diffuse striatal as well as cortical motor and premotor A-CBF increase during ictal examinations in GPDs associated with motor manifestations (jerks in SSPE) and focal CBF increase in C-S-T, C-T or S-T circuits in the presence of LPDs with or without motor manifestations (stroke-like events and asymmetrical ES) with straight topographical correlation with the EEG focus. During inter-ictal examinations, patients with drug-resistant ES disclosed A-CBF decrease in corresponding C-S-T, C-S, C-T or S-T circuits, which seemed related to age, more frequent after age 1 year. Another finding in ES group was R-CBF increase in corresponding S-T circuits, more frequent when compared with DRFE group, and not related to VGB treatment.

These findings suggest that GPDs and LPDs including ES, with or without clinical manifestations, involve C-S-T loops and their intrinsic functional properties. Based on EEG patterns and our ASL-MRI findings, we propose a model for the generation of PDs and of ES by combining existing pathophysiological models of C-S-T network dynamics.

### GPDs and periodic jerks in SSPE are correlated with diffuse striatal CBF increase, while LPDs with focal C-S-T CBF increase, topographically corresponding to the EEG focus

Here we report the first studies combining EEG and ASL-MRI in two patients with SSPE and WS, performed during inter-ictal and ictal states. Striatal, thalamic, and cortical motor and premotor CBF increase during GPDs and LPDs suggests an ictal mechanism of the CBF changes. Reversible striatal hypermetabolism on FDG-PET scan or hyperperfusion on ictal single photon-emission computed tomography (SPECT) were already reported in several CNS diseases such as SSPE, Sydenham’s chorea, primary antiphospholipid syndrome, neurosyphilis, leucine-rich glioma-inactivated 1 encephalitis and limbic encephalitis with subacute dementia, supposed being of auto-immune or inflammatory origin and/or related to movement disorder.^31–37^ However, striatal hypermetabolism was also described in non-auto-immune disorders such as Moya–Moya disease and hyperglycaemia.^38,39^ As hyperperfusion of basal ganglia (BG) has also been described in metabolic disorders,^40^ Leigh disease was initially suspected but not confirmed in our patients with LPDs. Using cortical, striatal and thalamic segmentation, we showed in our patients with ictal examinations, for the first time, A-CBF and R-CBF increase in corresponding C-S-T compartments, topographically correlated to the EEG focus, not necessarily related to motor manifestations neither to an auto-immune phenomenon. Moreover, changes in A- and R-CBF in corresponding C-S-T, C-S, C-T or S-T circuits was observed in ASL-MRI in patients with ES, in both ictal and inter-ictal state. Therefore, these striatal and thalamic CBF changes cannot be explained exclusively by a motor phenomenon involving BG. Thus, the CBF increase in the presence of PDs seems to reflect an ictal mechanism involving topographically aligned specific C-S-T circuits and supports the statement that PDs with clinical manifestations correspond to an ictal event.^1^

Generalized periodic discharges and periodic jerks (SSPE) corresponded to diffuse CBF increase in striatal structures allied with CBF increase in motor and premotor regions, whereas LPDs were related to a focal CBF increase in C-S-T circuits, topographically correlated to the EEG focus, involving motor regions when myoclonic jerks or asymmetric ES were present. Our findings therefore support the literature data that LPDs are rather triggered by a focal lesion, whereas GPDs are triggered by an abnormal input from rather large, multifocal cortical-subcortical lesions or genetic/chromosomic neurodevelopmental aetiologies.^41^ Depending on the extend of cortical-subcortical lesions, different proportions of motor or non-motor C-S-T circuits may be involved, ranging from localized C-S-T circuits associated with focal features to diffuse or generalized C-S-T circuits with massive motor manifestations. The semiology of motor manifestations (myoclonus versus spasm) during PDs could therefore depend on the amount of motor and premotor C-S-T circuits involved (myoclonus limited to motor C-S-T while ES involving motor and premotor C-S-T). This could also explain asymmetric motor manifestations reported in SSPE as well as in ES.^8,9,42^ The progressive alteration of background activity and periodicity changes with shortening of inter-GPD intervals in advanced SSPE reflect the progression of the disease, the extent of the lesions and a pejorative prognosis.^43^ Striatal and thalamic hyperperfusion concordant with the epileptogenic zone is also a common finding on ictal SPECT during focal seizures as demonstrated during frontal or temporal seizures with and without contralateral dystonic posturing.^44–46^ Synchronized cortico-striatal epileptic discharges have been recorded with precise temporal timing on stereo-EEG in focal seizures in adults.^47^ Thalamic, caudate nucleus and perirolandic hyperperfusion were also disclosed on ictal SPECT during myoclonic absences which are classified as generalized seizures.^48^ Our findings may reflect an ubiquitous ictal activation of C-S-T circuits. However, as disclosed using segmentation of inter-ictal ASL-MRI, C-S-T circuits are more frequently involved in patients with ES when compared with those with DRFE. We may hypothesize that the occurrence of the PDs rather than of focal seizures depends on the extent of excitatory input involving mainly cortical focal regions in focal seizures, and multifocal, diffuse and subcortical structures in PDs.

### ASL-MRI in patients with ES in inter-ictal state

The main observation in the ES group was decreased A-CBF in corresponding C-S-T, C-S, C-T or S-T circuits, observed mainly in patients >1 year old, thus possibly with longer disease duration. All ES disclosed highly abnormal inter-ictal EEG activity, suggesting its deleterious impact on CBF with CBF decrease in C-S-T circuits as a functional correlate. In contrast, thalamic A-CBF increase was observed in four patients, all <1 year old, however, not significantly related to age nor to VGB medication. Thus, thalamic A-CBF increase is likely related to the pathophysiology of ES. Moreover, the S-T, C-S or C-S-T R-CBF increase observed in 10/14 ES patients was not related either to VGB medication. When comparing ES and DRFE groups, CBF changes in topographically aligned C-S-T circuits were more frequent in ES group. Thus, C-S-T loops seem to be involved in the generation of PDs and of ES, but not in DRFE, despite daily seizures.

It is noteworthy, that in our control group, A-CBF values in the first year of life were about 50% higher in thalamic and striatal structures when compared with cortex (Supplementary Fig. 1B and C). This ratio decreased later to 1, due to the subsequent increase of cortical CBF related to the cortical maturation.^49^ This observation and the result of increased R-CBF in thalamic and striatal structures in our ES patients suggest the physiologically preponderant functional role of the basal midbrain structures at this age period and their possible enhanced impact on ictal phenomenology when compared with older children. The hypothesis that WS may result from pathological entrainment of physiological C-S-T loops is also supported by the fact that some infants with ‘idiopathic’ WS might have spontaneous and complete remission.^50^

Our ASL-MRI findings add new data to the literature. Relative striatal hypermetabolism on FDG-PET (values neither compared with controls nor using C-S-T segmentation) was reported in 32 of 44 children with WS of various aetiologies, independent of their age or sex, occurrence or absence of clinical ES and EEG pattern during the FDG-uptake period, and not related to aetiology.^23^ Striatal hyperperfusion with additional hyper- or hypoperfusion in cortex and cerebellum was also reported on ictal SPECT in 6/9 patients with WS.^51^ In the context of a focal cerebral lesion, clinical focal signs such as asymmetry or asynchrony of ES, associated focal motor, autonomic and oculomotor manifestations, are usually consistent with the topography of the lesion.^52^ Also, an inter-ictal EEG-fMRI study comparing patients with WS and focal epilepsy showed that blood oxygenation level-dependent (BOLD) changes differed by significant increase in putamen and brainstem in the WS group.^53^ These findings were corroborated by the same group using EEG source analysis (dynamic imaging of coherent sources) and showed that the slow delta activity in hypsarrhythmia was associated with coherent sources in the occipital cortex as well as in the parietal cortex, putamen, caudate nucleus and brainstem.^54^ Our study results neither provide temporal timing nor the direction of the activation of C-S-T loops as the ASL-MRI summarizes a 4 min period of neurovascular coupling (see Supplementary Material Methods). However, the assumption that the ictal discharge triggering the ES originates from the cerebral cortex (i.e. underlying lesion) is supported by data from the literature, from electrocorticography, high-frequency oscillation analyses during ES and by the fact that patients may completely recover after focal cortical surgery.^55–58^ Only three of our patients with ES had focal cortical dysplasia which involved however at least two cerebral lobes. All others were of genetic, chromosomal or post-infectious origin, or involved large or multifocal cerebral lesions, thus possibly explaining the large and multifocal CBF changes as well as diffuse abnormal input to BG.

Our EEG and ASL-MRI findings in patients with ES, GPDs and LPDs as well as the previously reported studies in patients with SSPE, GPDs and ES highly suggest that corresponding C-S-T circuits are involved in the generation of these electro-clinical patterns and that the ictal phenomenology results from the synchronization of cortical and thalamic loops modulated by BG.

### Proposed model: PDs with or without clinical manifestations result from the activation of C-S-T loops, their topographic input and output, and from the physiological properties of the thalamic and basal ganglia neurons (Fig. 7)

The possibility of neurological (positive and negative) manifestations resulting from the dysfunction of corticothalamic circuits modulated by BG was conceptualized by Llinas and Steriade under the term of ‘thalamic dysrhythmia syndrome’^59^ and recently demonstrated in invasive EEG recordings in children with focal epilepsy.^60^ A recent study with a data-driven approach using support vector machine learning, analysing resting-state EEG oscillatory patterns in patients with Parkinson’s disease, neuropathic pain, tinnitus and depression, supported the existence of thalamic dysrhythmia (TD) as a mechanism underlying these neuropsychiatric disorders.^61^ TD is described as a state of imbalance of excitatory and inhibitory signals within the thalamocortical network. An abnormal input from the cortex (removal of excitatory information) may conduce the thalamic neurons to fire in the bursting mode out of physiological context of sleep or drowsiness called ‘disfacilitation’ or ‘deafferentation’.^62,63^ Thalamic bursting mode can also be related to protracted inhibition due to excessive inhibitory input from globus pallidus or reticular nucleus.^59,64^ In our patients with PDs/ES, the abnormal focal or multifocal cortical input to BG and thalamus is supported by the abnormal background EEG, focal slowing in patients with LPDs, diffuse slowing or depression in patients with GPDs as in SSPE or diffuse slowing (hypsarrhythmia) in ES in WS, thus facilitating hyperpolarization of cortical and thalamic neurons which may enter in the bursting mode (see Supplementary Material 1). Remarkably, background activity observed in-between PDs and ES does not correspond neither to wake nor to sleep EEG, and could represent EEG expression of TD.

Another argument for TD being involved in the C-S-T loops activation is its ‘facilitation’ during drowsiness.^59^ Interestingly, a characteristic feature of ES is their relationship with sleep-wake cycle occurring frequently at awakening or drowsiness. It is noteworthy that the mean age at onset of WS (6 months) coincides with the onset of a peculiar transitional EEG aspect at drowsiness and awakening, hypnagogic hypersynchrony, consisting in diffuse high-amplitude synchronous slow waves.^65^ This may suggest that at this age period, the thalamocortical synchronization undergoes a maturational progress.

Beside concomitant motor or non-motor periodic manifestations, PDs/ES show a typical EEG pattern consisting in stereotyped, time-locked, polyphasic, localized or diffuse waveforms that may be superposed with rapid rhythms (RRs), so-called PDs-plus, similar to sleep K-complexes, suggesting the involvement of large scale cortical and subcortical structures.^1,59,66^ Following the TD model, we can hypothesize that PDs and the concomitant activation of C-S-T circuits, we observed might result from an abnormal synchronization of thalamocortical networks modulated by BG. As in PDs-plus, the ictal ES EEG pattern may include RR following the initial negative slow wave at its descending slope.^9,67^ The beginning of this descending slope precedes the ES EMG onset by an interval of 182 ± 127 ms.^67^ This EEG/EMG correlation was similar in our patient with SSPE consisting in left central-parietal negative sharp wave, which also preceded the right deltoid contraction with a latency longer than expected for corticospinal conduction. The morphology and polarity of the ictal slow wave of ES, as well as the frequent coalescence with RR are consistent with recordings of the transition from the silent state (hyperpolarizing) or downstate to the active state (depolarizing) or upstate of thalamocortical neurons, characteristic of the thalamocortical synchronization in non-rapid eye movement (NREM)-sleep and the thalamic ‘bursting mode’^68,69^ (Supplementary Material 1). Indeed, as described by Steriade and Amzica, cortical ‘down state’, thus hyperpolarization corresponds to the surface EEG negative slow wave, whereas the ‘up state’ corresponds to the cortical positive slow wave. They also demonstrated that the intracortical coherency of fast oscillations is coupled with synchronized RR in corticothalamic circuits during the active state.^70^ The RR reflect synchronized high-frequency bursts of spikes during the active state corresponding to the activation of thalamocortical neurons, and could theoretically generate motor manifestations, when motor thalamus inputs/outputs are concerned.

**Figure 7:**
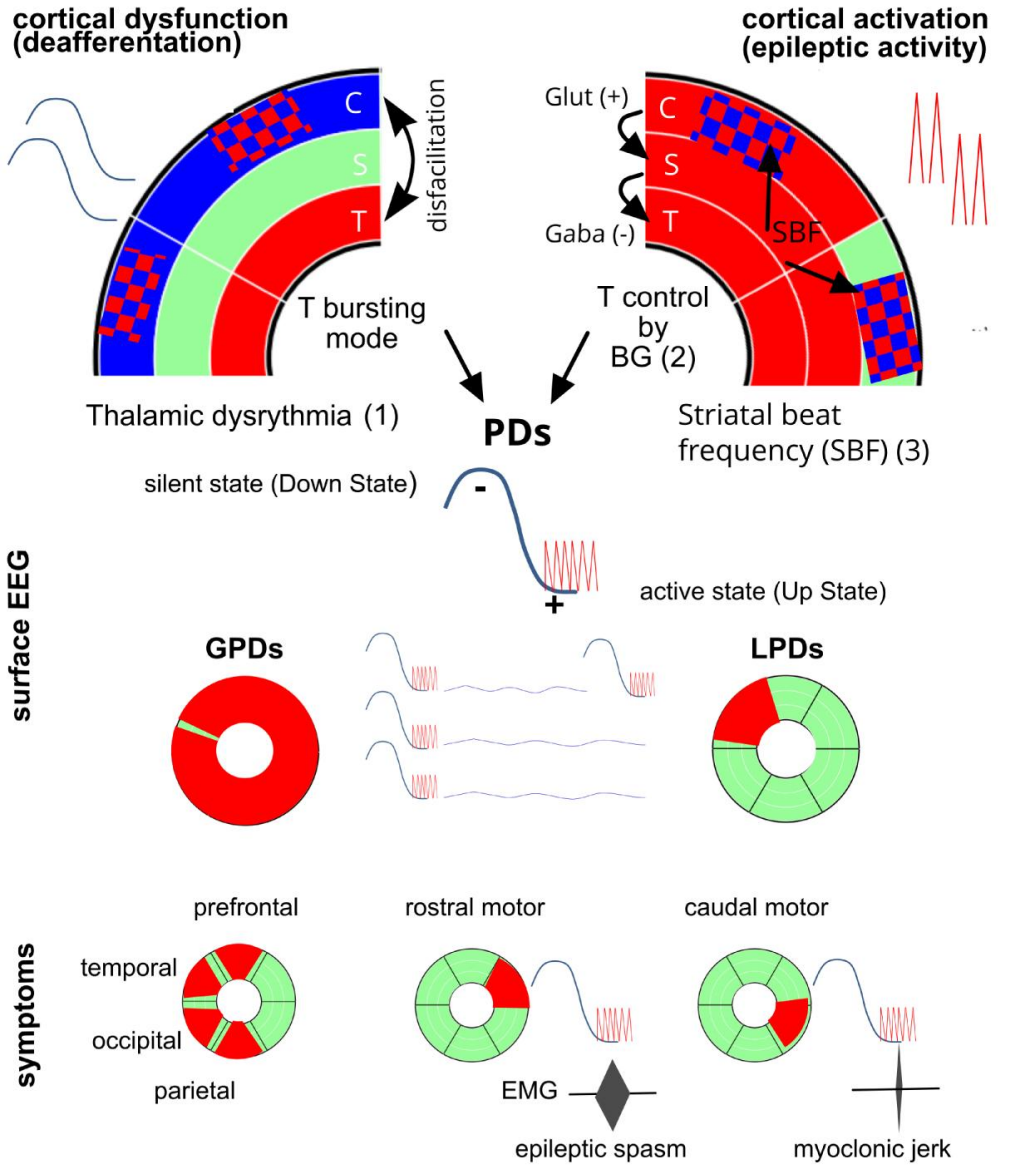
**Schema outlining the hypothesis of the generation of periodic discharges and epileptic spasms.** (1) Thalamic dysrhythmia (TD): abnormal cortical input due to focal, multifocal or diffuse cortical dysfunction conduces thalamic neurons to fire in the bursting mode out of physiological context of sleep or drowsiness called ‘deafferentation’ or ‘disfacilitation’.^59^ (2) Cortical epileptic/excitatory discharges provide glutamatergic input to the striatum leading to inhibitory basal ganglia impact on the thalamus (rebound model^60^) reinforcing the TD and/or disinhibiting the thalamic nuclei during the active state (upstate) of TD. Morphology and polarity of the ictal slow wave as well as the frequent coalescence with RRs are consistent with recordings of the transition from the silent state (hyperpolarizing) or downstate to the active state (depolarizing) or upstate of thalamocortical neurons, characteristic of the corticothalamic synchronization in NREM-sleep and the thalamic ‘bursting mode’ ^61,62^ (Supplementary Material 1). (3) The periodicity of periodic discharges (PDs) is facilitated by the SBF model^61^ applied to a context of epileptic/paroxystic input of cortical activity to the striatum (see Supplementary Material 2). The ictal phenomenology (motor or non-motor) during the active states (upstates) of TD depends on localization and extend of the cortical excitatory input and on the amount of C-S-T circuits involved in the ictal discharge. C cortex; S striatum; T thalamus; red CBF increase, blue CBF decrease; GPD generalized periodic discharges; LPD lateralized periodic discharges.

The different ways of motor thalamus nuclei control by BG were conceptualized by Bosch-Bouju and colleagues^71^ proposing three main mechanisms: the rebound model focusing on the BG ability to inhibit thalamus, thus triggering low-threshold-calcium-spike bursts, the gating model activating thalamus through disinhibition (via the direct pathway inhibiting BG output nuclei which at rest or without the cortico-striatal input to the BG output nuclei inhibit thalamus), and the entrainment model focusing on the timing of BG inputs (Supplementary Material 2). In our SSPE and ES patients, the cortical input on thalamus may be considered dual: the slowing of the background activity due to cortical dysfunction, thus the ‘deafferentation’, may induce TD; besides, cortical epileptic/excitatory discharges provide glutamatergic input to the striatum leading to inhibitory BG input to thalamus (rebound model) reinforcing the TD and the thalamic bursting mode (Fig. 7). The excitatory input from the ictal discharge, in this context, limited to the time window of the active state of TD could therefore explain short lasting ‘positive manifestations’ during ES and during PDs. Indeed, when acting on motor thalamus nuclei as well as on the superior colliculus, excitatory cortical input could theoretically provoke limb jerks and brief eye movements^68,72^ (Fig. 7). Focal seizures may be associated with ES (precede or follow), which could be explained by different models of BG action on thalamus.^71,72^

Motor and non-motor features of PDs and of ES could therefore result from the cortical excitatory input/activation of topographically aligned corresponding striatal and thalamic segments modulated by BG during the active states of slow-wave oscillations during TD. The activation of ES at awakening may be due to increased cortical excitability (during excitation-inhibition imbalance) due to the forebrain arousal systems from the brainstem cholinergic pedunculopontine tegmental nucleus facilitating the depolarization of thalamocortical neurons.^68,73^ The hypothesis of the implication of the thalamocortical networks in the pathophysiology of the WS is supported by the enhanced thalamocortical connectivity as well as ictal increased BOLD signal in brainstem, BG and thalamus as demonstrated by fMRI studies in Lennox–Gastaut syndrome which often follows drug-resistant WS and is considered as its age-dependent continuum.^74–76^ Additionally, the periodicity of PDs and ES could be favoured by cortico-striatal network properties as conceptualized in the striatal beat frequency (SBF) model^77^ applied to a context of epileptic input of cortical activity to the striatum (see Supplementary Material 2). Multifocal epileptic discharges may be encoded by striatal medium spiny neurons (MSNs) that detect coincident activity, and via the SBF model synchronize the ictal activity to MSNs ‘to be timed’ signal.^77–79^ The observation that periodicity changes during SSPE disease progression with shortening of inter-GPD intervals could be explained by higher occurrence of pathological synchronicity and therefore more frequent coincident activity detection in the cerebral cortex due to progressive neuronal loss compared with a normal cortex with higher complexity and variability.

Our model remains highly speculative and lacks demonstration of precise temporal timing of the interaction in C-S-T circuits. However, it attempts to connect EEG and clinical features with well-documented models of thalamocortical networks and their modulation by BG. Also, our findings may be considered to reflect a basic epileptic process implying cortico-striatal and thalamic loops as previously conceptualized in focal and absence seizures.^47,80,81^ However, epileptic processes in PDs and ES are different from those in absence seizures. Clinically, PDs and ES are accompanied by various motor and non-motor manifestations depending of their cortical topography while in absence seizures the main manifestations are motor arrest, impairment of awareness and eventually mild motor manifestations and/or automatisms.^82^ ES may follow, precede or occur simultaneously with focal seizures suggesting that, regarding cortico-striatal-thalamic interactions, the mechanism in ES is rather in line with those described in focal seizures where rapid discharges have been recorded in the cortex and in the striatum at the onset of the epileptic discharge.^47^ The difference in C-S-T interactions in focal versus absences seizures is also supported by human EEG-fMRI studies showing BOLD signal increase in the medium thalamus but decrease in caudate nucleus in absence seizures while the BOLD signal as well as the CBF are increased in both thalamus and BG during the propagation of focal seizures.^83–88^ In our patients, ASL-MRI showed clearly increased neurovascular coupling in striatal structures during PDs and ES suggesting that the striatal output neurons are rather not inhibited contrasting with the BOLD signal decrease described during absence seizures. The fact that antiseizure drugs increasing GABA levels such as VGB aggravate or even induce absence seizures and in contrast are effective in ES, also corroborates a different interaction within C-S-T loops in absence seizures compared with ES.^89–91^ VGB may act by increasing the inhibitory input of the thalamocortical GABA-ergic neurons, but it probably also acts by decreasing the glutamatergic excitatory input from the cortex to the BG, thus interrupting the C-S-T loops, hypothesized to be involved in ES. The effect of VGB on the BG in WS is supported by the fact that around 25% of children with WS treated with VGB disclose reversible MRI signal changes in thalamus, BG, brainstem, tegmentum and cerebellar nuclei.^92^ Furthermore, some children develop movement disorders under VGB, related or not to these MRI changes.^93,94^ In our ES group, 9 of the 11 patients showing R-CBF changes in striatum and thalamus received VGB at the time of ASL-MRI, but no one showed anatomical signal changes in BG or thalamus. In addition, four of the five patients without VGB treatment disclosed R-CBF increase in thalamus and striatum supporting that these CBF changes were not related to VGB treatment.

### Limitations and future directions

There were several limitations in our study with the first and foremost its retrospective design and, due to the rare pathology of patients with periodic EEG patterns, the small number of patients in this group. Moreover, ages in ES and DRFE groups differ with few older children in ES and few younger children in DRFE. Most of our younger patients were recorded in sleep, either natural or sedation induced; however, the correspondent controls were also matched for sedation status during MRI examinations. Also, most patients in our study received various ASM, possibly affecting brain activity. Although ASL-MRI shows an excellent spatial resolution, this technique does not provide insights in the dynamics or temporal interactions between the investigated structures. Moreover, cerebellar perfusion was not evaluated, which should be realized in the future, as cerebellum is highly involved in BG-thalamus interplay.

## Conclusion

We show for the first time that specific C-S-T circuits are involved in PDs with and without motor manifestations, including ES. We hypothesize that PDs (LPDs, GPDs) result from the pathologic synchronization of C-S-T circuits and their discharges. Based on these findings, we propose a hypothesis for the generation of PDs and of ES resulting from the combination of previously described models of C-S-T circuit dynamics. Our findings open new insights into the pathophysiology of PDs and ES. Our proposed model, based on a disturbed balance of specific C-S-T circuits involved, may open new therapeutical perspectives in ES.

## Supplementary Material

fcac250_Supplementary_Data

## Data Availability

The datasets generated during and/or analyzed during the current study are available within the article and its supplementary material. Additional anonymized data are available from the corresponding author on reasonable request.

## Abbreviations

A-CBF =: absolute CBF
ASL =: arterial spin labelling
ASM =: antiseizure medication
BG =: basal ganglia
C =: cortex/cortical
CAT =: computational anatomy toolbox
CBF =: cerebral blood flow
CNS =: central nervous system
DRFE =: drug-resistant focal epilepsy
ES =: epileptic spasms
GPDs =: generalized periodic discharges
LPDs =: lateralized periodic discharges
MSNs =: medium spiny neurons
NREM =: non-rapid eye movement (sleep)
PDs =: periodic discharges
R-CBF =: ratio CBF
ROI =: regions of interest
RRs =: rapid rhythms
S =: striatum/striatal
SBF =: striatal beat frequency
SPECT =: single photon-emission computed tomography
SPM12 =: statistical parametric mapping 12
SSPE =: subacute sclerosing panencephalitis
T =: thalamus/thalamic
TD = =: thalamic dysrhythmia
VGB =: Vigabatrin
WS =: West syndrome
