# Supplementary material for: Periodic electroencephalographic discharges and epileptic spasms involve cortico-striatal-thalamic loops on Arterial Spin Labeling Magnetic Resonance Imaging
Source: Brain Commun. 2022 Oct 6;4(5):fcac250. doi: 10.1093/braincomms/fcac250 (PMC9598541; doi:10.1093/braincomms/fcac250)
Supplement: fcac250_Supplementary_Data [file fcac250_supplementary_data.zip › Supplementary Table 1.docx]

**Supplementary Table 1 Patients and electro-clinical characteristics**

| **Patient/ exam n°** | **Age at**  **ASL-MRI** | **Etiology** | **Age at seizure onset** | **Seizure type(s),**  **Epilepsy/epilepsy syndrome** | **EEG** | **MRI** | **Drug at exam** |
| --- | --- | --- | --- | --- | --- | --- | --- |
| **Groupe 1 - ASL-MRI in both interictal and ictal states** | | | | | | | |
| 1a (interictal) | 14y7m | SSPE | 14y | None | No GPD | R O Fl and diff HI  no BG nor T anomaly | 0 |
| 1b  (ictal) | 14y7m | SSPE | 14y | PS | GPD | Middle cerebellar peduncle Fl and diff HI | 0 |
| 1 (ictal) | 15y2m | SSPE | 14y | PS | GPD | Bilateral putamen and caudate HI, no T nor BS anomaly | 0 |
| 26a  (interictal) | 6m | Cortical malformation | 4,5m | aES | HA, temporal Sp SpW SlW bilateral | L extended posterior and R Temp signal abnormalities | VGB (m2), CLB, TPM, CBZ, Nsed |
| 26b (ictal) | 6m | Cortical malformation | 4,5m | aES | Ictal EEG (ES): L T FR bursts |  | VGB (m2), CLB, TPM, CBZ, Nsed |
| **Group 2 (ictal) – Periodic Discharges** | | | | | | | |
| 2 | 0y6m | Stroke-like episode, de novo SCN1A mutation | 5m | FCS SE | R hem (CT) PDs at 1 Hz, L FC PD (of lower amplitude) + right-sided clonus | Fl and Diff HI in bilateral peri-rolandic and insular cortex, bilateral pallidum and putamen (VGB?) and R T  global supratentorial atrophy | LEV, VGB (d41), CZP, KD, Nsed |
| 3 | 12y5m | Stroke-like episode of N.I. etiology | 11y | FCS SE | L hem (FC) PDs at 1.5 Hz, eyelid myoclonia | L T Fl HI | VPA, LEV, CZP |
| 4a | 11y11m | Stroke-like episode of N.I. etiology | 11y | BFCS SE | 26/10/12 (24h): R>L FC PD at 1 Hz/no motor manifestations | R Temp and R frontal mesial insular Fl and Diff HI | VPA, LEV, LTG, KD |
| 4b | 13y3m | Stroke-like episode of N.I. etiology | 11y | BFCS SE | L hem PD at 1.5 Hz, posterior predominance, R LL clonus | L TempO insular and left T T2 and Diff signal abnormalities | VPA, LEV, LTG, KD |
| **Group 3 (interictal) – Epileptic Spasms/West Syndrome** | | | | | | | |
| 5 | 8m | T21 | 6m | ESc, WS | HA | Fl and Diff HI in bilateral pallidum and BS nuclei (VGB?)  global supratentorial atrophy | VGB (m2), HC, ACTH  Nsed |
| 6 | 7m | N.I. | 7m | ESc, WS | HA | nl | 0, Nsed |
| 7 | 1y6m | Herpes encephalitis at age 15 m | 1y6m | ESc, FE, LoES | EE, R>L TempO SlW/Sp | Major supra- and infratentoriel atrophy, bilateral TempP and F signal anomalies, periventricular white matter anomalies | VPA, Nsed |
| 8 | 6m | Cortical malformation | 6m | ESc, FBAS, WS | HA, R T SlW/Sp | R FCP gyration anomaly | VGB (d1), Nsed |
| 9 | 6y10m | N.I. | 6m | ESc, MS | EE, bi-O, FT SlW/SpW | nl | 0  Sed (4.5 ml PB) |
| 10 | 7m | N.I. polymalformation syndrome | 6m | ESc, WS | Atypical HA, R TempO SlW | ventricular dilatation, minor supra- and infratentoriel atrophy | VGB (w3), HC  Sed (1.4 ml PB) |
| 11 | 6m | N.I. | 5m | ESc, WS | HA, L TempO SlW | Probable R TempO gyration anomaly | VGB (d2), Nsed |
| 12 | 5y6m | Auto-immune encephalitis of N.I. etiology | 5y (TS), 5y5m (asES) | BFCS, asESc | EE, multifocal Sp | R peri-rolandic and mesial cortex Fl HI | VGB (w6), HC, KD |
| 13 | 9m | T21 | 9m | ES, WS | HA | nl | 0, Nsed |
| 14 | 8m | Cortical Malformation | 8m | aES, WS | HA, L CT Sp | L polymicrogyria | VGB (d2)  Sed (2 ml PB) |
| 15 | 7m | Prematurity 36GW | 6m | ES, WS | HA, L>R TempO Sp | nl | VGB (d10), Nsed |
| 16 | 8m | Dup15q | 8m | ES, TS, WS | Atypical HA, diffuse FR | Minor retardation of myelination | VGB (d4), Nsed |
| 24 | 3y4m | Cortical malformation | 8m | ES | Post occ rhythm 7 Hz, bilateral SlW/S focus with R Templ predominance | Large R F cortical malformation | VPA, LTG, HC (m1), KD (m2), Sed (8ml PB) |
| 25 | 1y11m | Cortical malformation | 2m | ES | HA in sleep, left PO SW/S focus | Extended L TempP cortical malformation | Vimpat, VGB (m17), CBZ, KD, Nsed |
| **Group 4 (interictal) – Drug-resistant Focal Epilepsy** | | | | | | | |
| 17 | 15y7m | L F dysplasia (IIb) | 7y | Focal motor with ictal pouting | nl BackG, L FC SlW,  L FC Sp/SpW bursts | L mesial superior frontal gyrus dysplasia | CBZ, LCM |
| 18 | 13y9m | R F dysplasia (IIa) | 12y | Focal aware non motor seizures | nl BackG, R F SlW/Sp | R supplementary motor area dysplasia | CBZ |
| 19 | 9y11m | R insular dysplasia (histology neg) | 4y | Hypermotor seizures | nl BackG, R FT SlW/Sp/FR bursts | R insular dysplasia | KD |
| 20 | 10y4m | R Temp dysplasia (histology neg) | 4y | Focal aware non motor seizures | nl BackG, R T SlW | R Temp dysplasia | LEV, VPA |
| 21 | 13y3m | R fronto-operculo-insular dysplasia (IIb) | 5,5y | FMS without clear-cut lateralisation | nl BackG, R F SlW/Sp | R fronto-operculo-insular dysplasia | VGB, CBZ |
| 22 | 6y9m | L F dysplasia (Ic) | 3,5y | FS (axial myoclonia followed by asymmetrical tonic arm contraction) | nl BackG, L F SlW/Sp | L F dysplasia | uVPA, LTG |
| 23 | 7y9m | R P dysplasia (IIa) | 5y | Focal motor (L hem tonic), automatisms | nl BackG, R PO SW/Sp | R P dysplasia | CBZ, LEV |
| 27 | 6m | TSC | 6wk | R occ subclinical discharges | nl BackG, R O Sp | Cortical and sub-cortical tubers predominating in F areas, also in R P, bilateral SEGA, sub-ependymal nodules | VGB (m5)  Sed (1.4 ml PB) |

**Abbreviations:**

Age/duration: d days; m months; wk weeks; y years.

Etiology: GW gestational weeks; N.I. not identified; SSPE subacute sclerosing panencephalitis; T21 trisomy 21; TSC tuberous sclerosis complex.

Seizure types: BFCS bilateral focal clonic seizure; ES epileptic spasms; aES asymmetrical ES; ESc epileptic spasms in clusters; FMS focal motor seizure; FS focal seizure; FCS focal clonic seizure; FBAS focal behavior arrest seizure; LL lower limb; LoES late onset ES; MS myoclonic seizure; PS periodic spasms; SE status epilepticus; TS tonic seizure.

EEG: nl BackG normal background; EE EEG of epileptic encephalopathy; FR fast rhythms; GPD generalized periodic discharge; HA hypsarrhythmia; LHPD left hemispheric periodic discharge; PD periodic discharge; sim simultaneous; SlW slow waves; Sp spikes; SpW spike waves.

MRI: HI hyperintensities; Fl Flair; Diff diffusion; BG basal ganglia; BS brain stem; T thalamus; SEGA subependymal giant cell astrocytoma

Localisation: L left; R right; Hem hemispheric; F frontal; C central; P parietal; O occipital; Temp temporal; TempO temporo-occipital; TempP temporo-parietal; FCP fronto-centro-parietal.

Drugs: CBZ Carbamazepine, CLB Clobazam, CZP Clonazepam, KD Ketogenic diet, LEV Levetiracetam, LCM Lacosamide, LTG Lamotrigine, PB pentobarbital (25mg/ml) STP Stiripentol, TPM Topiramate, VGB Vigabatrin (treatment duration in d days, w weeks, m months); VPA Valproic acid, ZNS Zonisamide. NSed no sedation, Sed sedation.
