# Supplementary material for: Periodic electroencephalographic discharges and epileptic spasms involve cortico-striatal-thalamic loops on Arterial Spin Labeling Magnetic Resonance Imaging
Source: Brain Commun. 2022 Oct 6;4(5):fcac250. doi: 10.1093/braincomms/fcac250 (PMC9598541; doi:10.1093/braincomms/fcac250)
Supplement: fcac250_Supplementary_Data [file fcac250_supplementary_data.zip › Supplementary_Material_including_Supplementary_Figures.docx]

**Supplementary Material**

**Periodic EEG discharges and epileptic spasms involve cortico-striatal-thalamic loops on ASL-MRI**

Monika Eisermann^1,¥^, Ludovic Fillon^2,3,4¥^, Ana Saitovitch^2,3,4^, Jennifer Boisgontier^2,3,4^, Alice Vinçon-Leite^2,3,4^, Volodia Dangouloff-Ros^2,3,4^, Thomas Blauwblomme^5^, Marie Bourgeois^5^, Marie-Thérèse Dangles^1^, Delphine Coste-Zeitoun^1^, Patricia Vignolo-Diard^1^, Mélodie Aubart^6^, Manoelle Kossorotoff^7^, Marie Hully^7^, Emma Losito^1^, Nicole Chemaly^8^, Monica Zilbovicius^2,3,4^, Isabelle Desguerre^6^, Rima Nabbout^8^, Nathalie Boddaert^2,3,4¥^, Anna Kaminska^1,¥^

**Author affiliations :**

^1^ Clinical Neurophysiology, Hôpital Necker Enfants Malades, AP-HP, Paris Université, Paris France

^2^ Pediatric Radiology Department, AP-HP, Hôpital Necker Enfants Malades, Université de Paris, F-75015, Paris France

^3^ Université de Paris, Institut Imagine INSERM U1163, F-75015 France

^4^ U1299 Trajectoires développementales & psychiatrie

^5^ Pediatric Neurosurgery, Hôpital Necker, APHP, Paris France, Université de Paris, Paris, France, INSERM U1163, IHU Imagine, Paris, France

^6^ Pediatric Neurology Department, Hôpital Necker Enfants Malades, AP-HP, INSERM U1163, Paris Université, Institut Imagine, Paris France

^7^ Pediatric Neurology Department, Necker Enfants Malades Hospital, AP-HP, Paris Université, Paris France

^8^ Reference center for rare epilepsies, department of pediatric Neurology, member of EPICARE network, institute Imagine INSERM 1163, Université de Paris, Paris, France

**Supplementary Figure 1A.**

Histogram showing the distribution of patients and controls by age range and scanner.


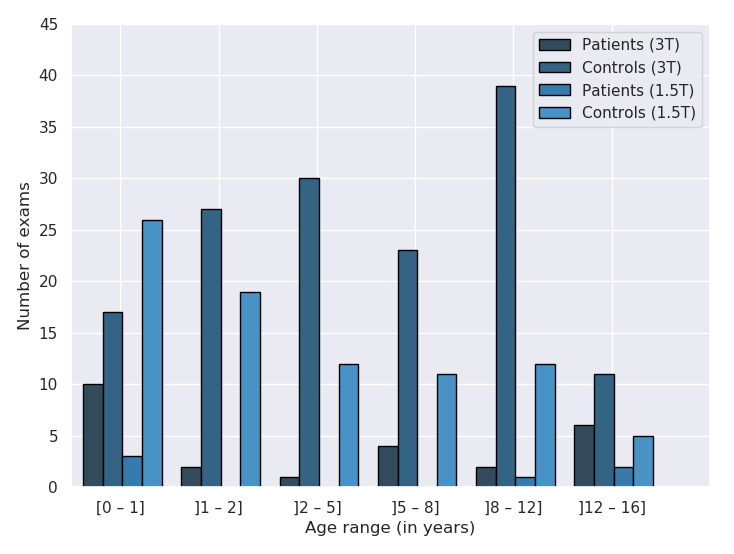


**Supplementary Figure 1B.**

**CBF – Ictal**





**Supplementary Figure 1B:** Scatterplots of the rest A-CBF (in mL/100 g/min; figure 3A) and the rest R-CBF (in arbitrary units; figure 3B) by scanner (1.5T and 3T) and by ROI (cortex, striatum, thalamus) with six measurements, per child and per plot, corresponding to the prefrontal, rostral and caudal motor, parietal, occipital and temporal sub-division for patients (red dots for ES, green dots for FE, blue dots for SSPE and orange dots for LPDs) and controls (grey dots) between 0 and 16 years. Patients were in ictal state during the ASL-MRI exam.

**Supplementary Figure 1C.**

**CBF – Inter-ictal**

**

**

**Supplementary Figure 8:** Scatterplots of the rest A-CBF (in mL/100 g/min; figure 4A) and the rest R-CBF (in arbitrary units; figure 4B) by scanner (1.5T and 3T) and by ROI (cortex, striatum, thalamus) with six measurements, per child and per plot, corresponding to the prefrontal, rostral and caudal motor, parietal, occipital and temporal sub-division for patients (red dots for ES, green dots for FE, blue dots for SSPE and orange dots for LPDs) and controls (grey dots) between 0 and 16 years. Patients were in inter-ictal state during the ASL-MRI exam.

**Supplementary Figure 2.**

**SUBACUTE SCLEROSING PANENCEPHALITIS – inter-ictal/ictal**

A-CBF inter-ictal (Right / Left)


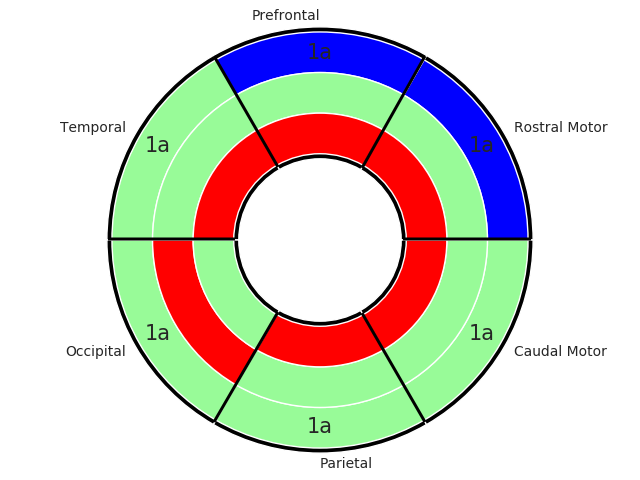

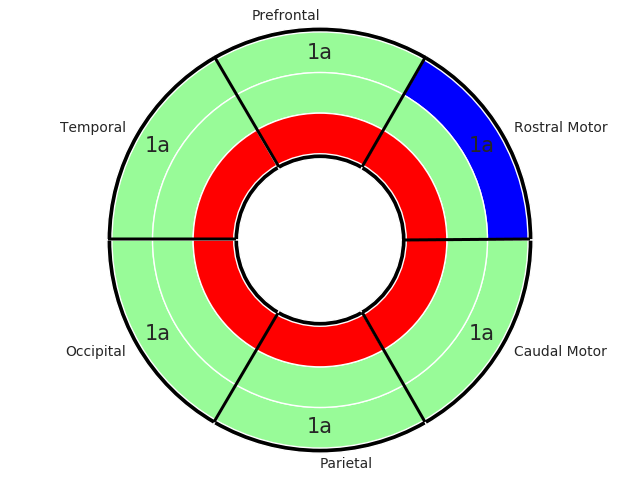


A-CBF ictal (Right / Left)


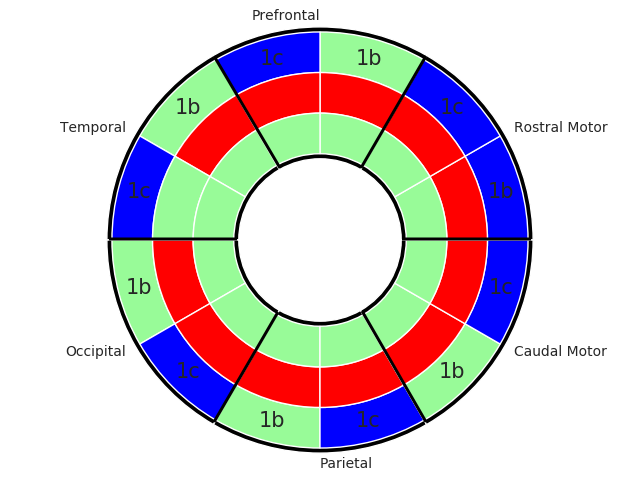

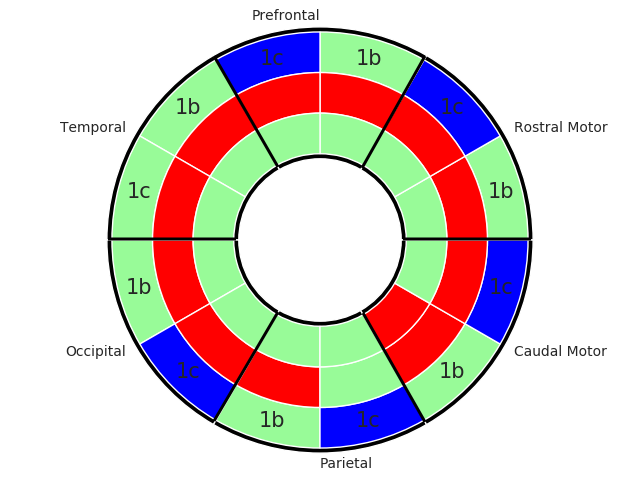


R-CBF inter-ictal (Right / Left)


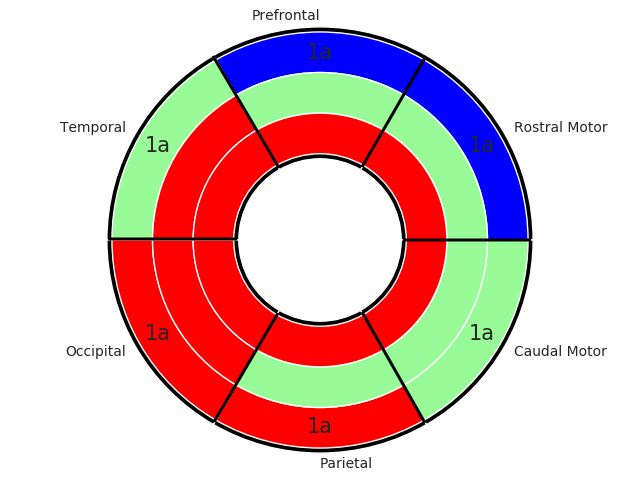

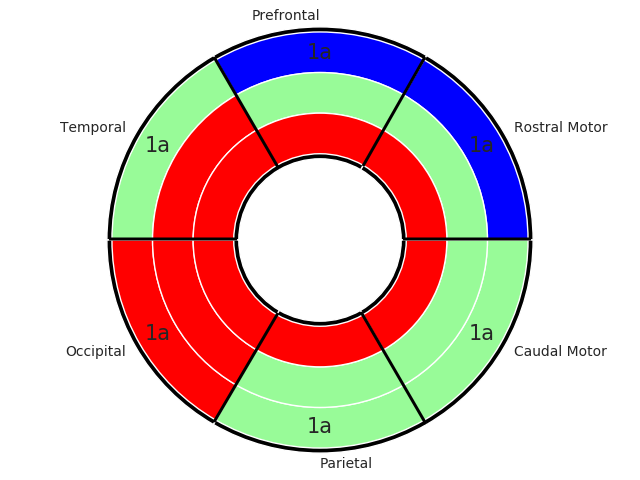


R-CBF ictal (Right / Left)


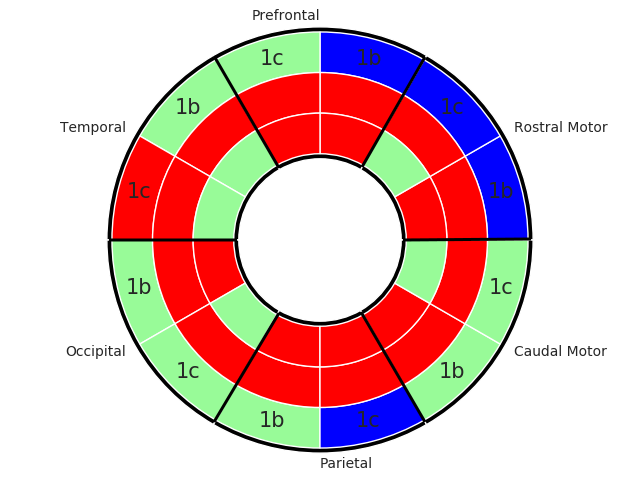

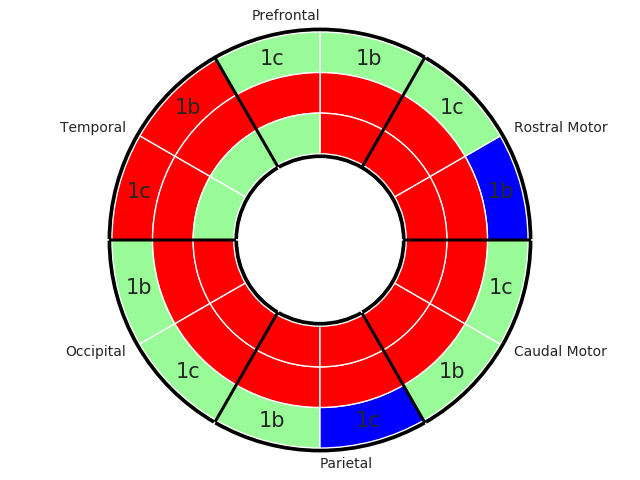


**Supplementary Figure 3.**

**EPILEPTIC SPASMS – inter-ictal/ictal**

A-CBF inter-ictal (Right / Left)


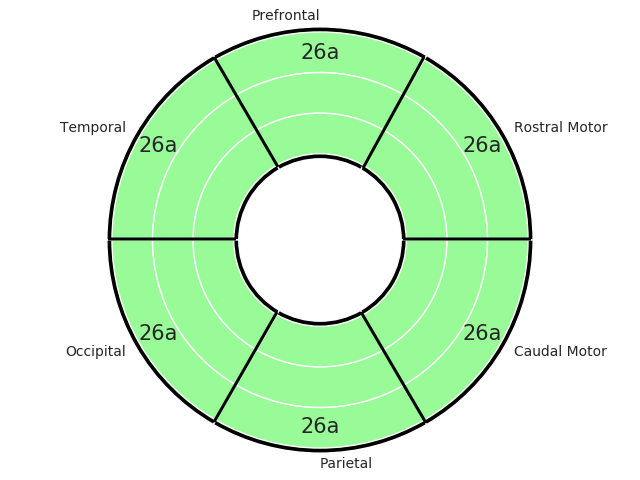

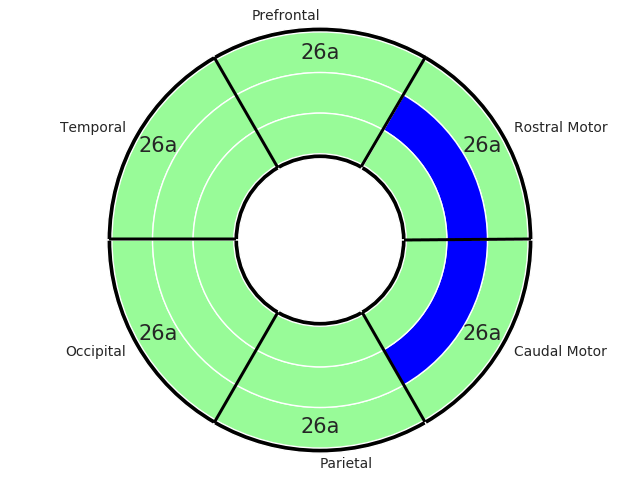


A-CBF ictal (Right / Left)


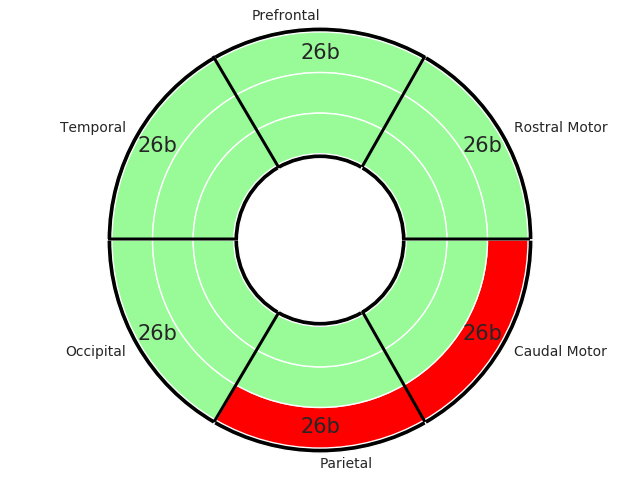

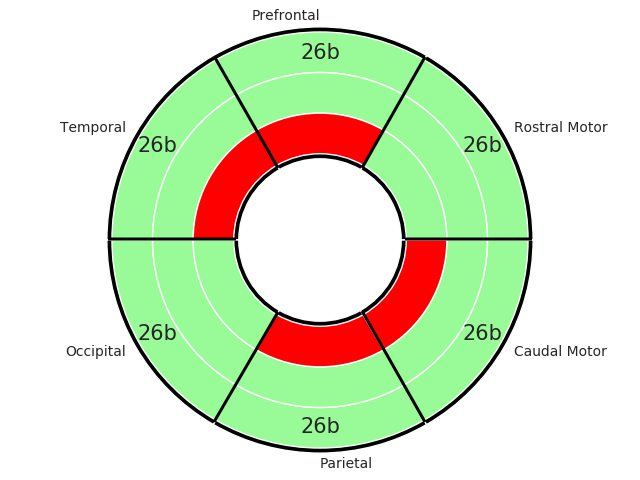


R-CBF inter-ictal (Right / Left)


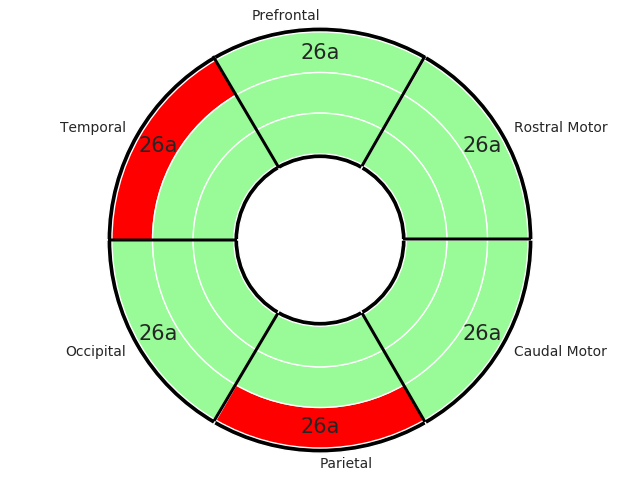

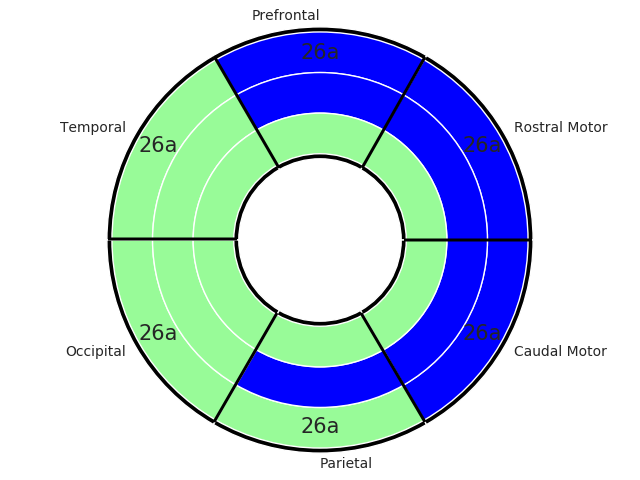


R-CBF ictal (Right / Left)


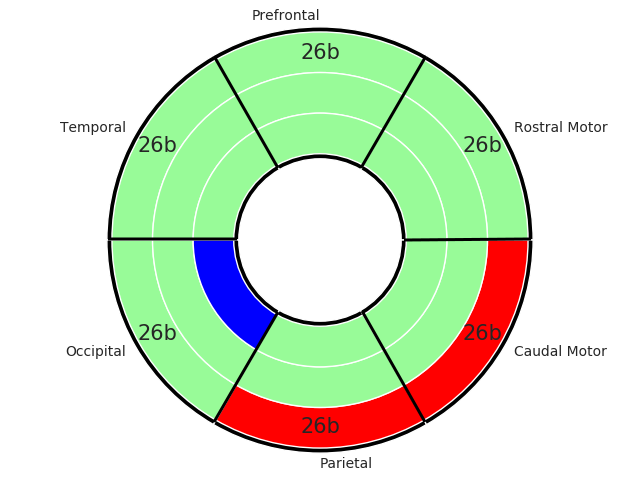

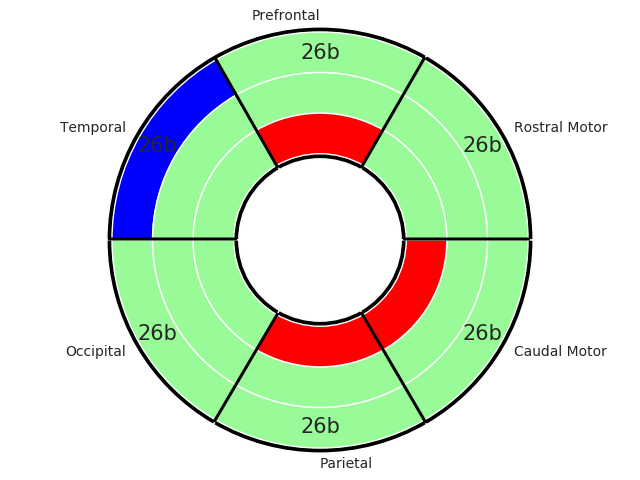


**Supplementary Figure 4.**

**PERIODIC DISCHARGES – ICTAL**

A-CBF ictal (Right / Left)

**
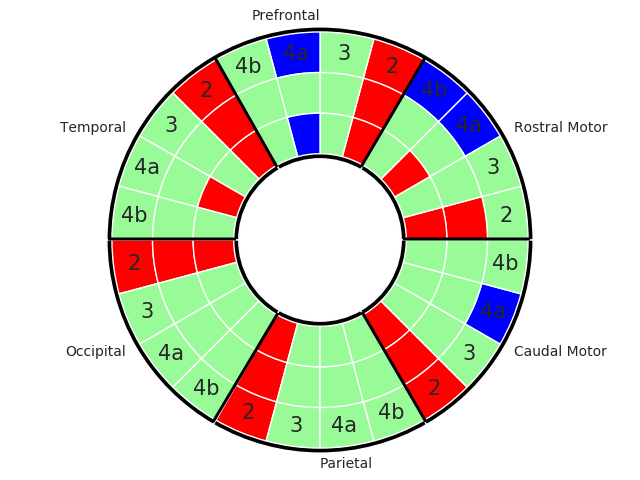
**
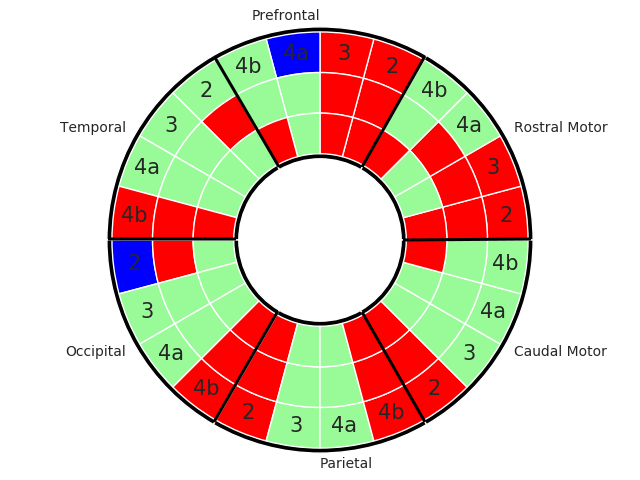


R-CBF ictal (Right / Left)


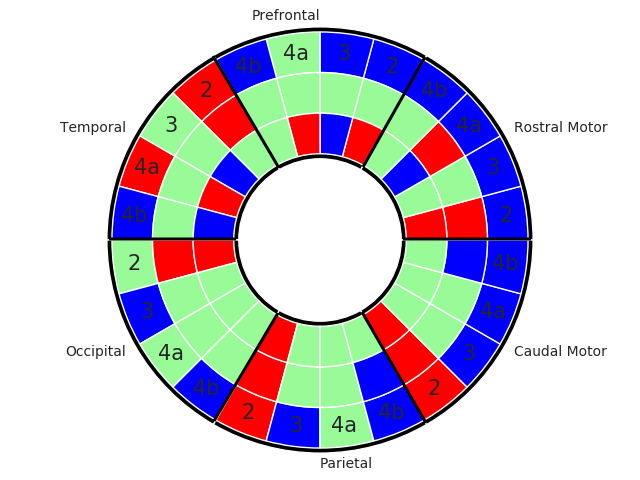

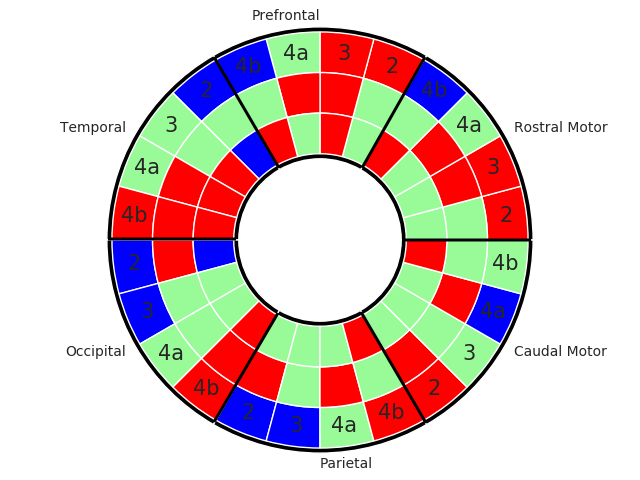


**Supplementary Figure 5.**

**EPILEPTIC SPASMS – INTER-ICTAL**

A-CBF inter-ictal (Right / Left)


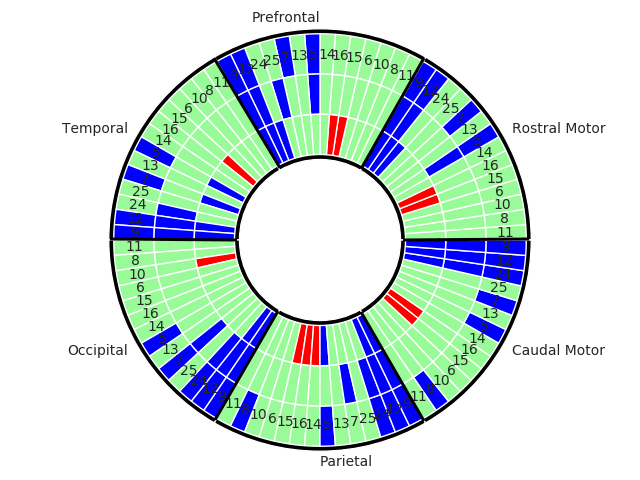

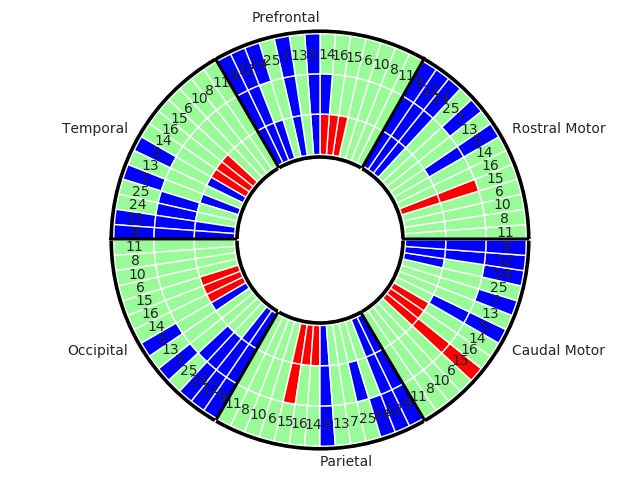


R-CBF inter-ictal (Right / Left)


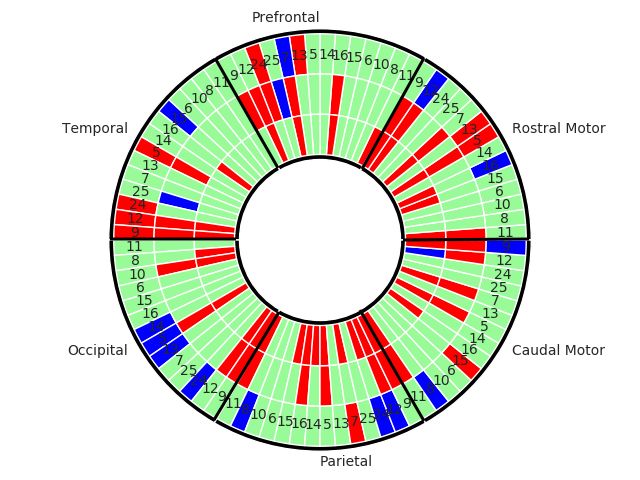

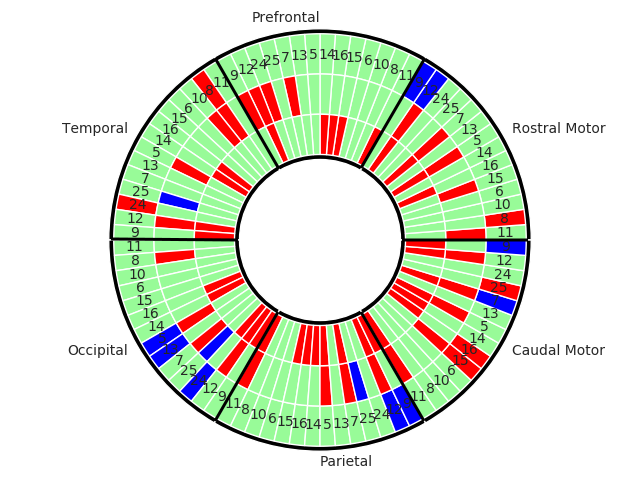


**Supplementary Figure 6.**

**FOCAL EPILEPSY – INTER-ICTAL**

A-CBF inter-ictal (Right / Left)

**
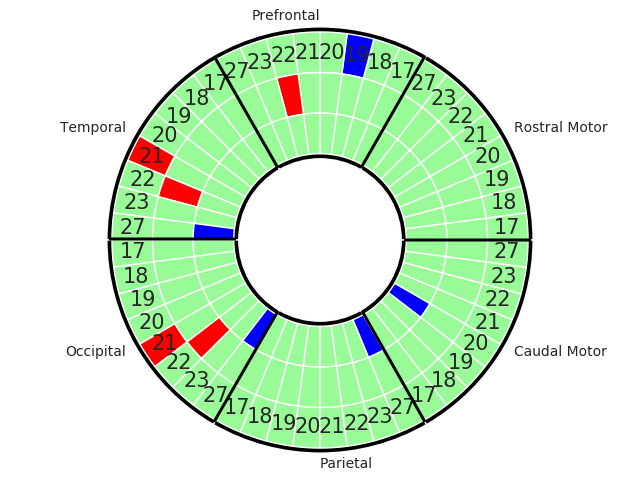
**
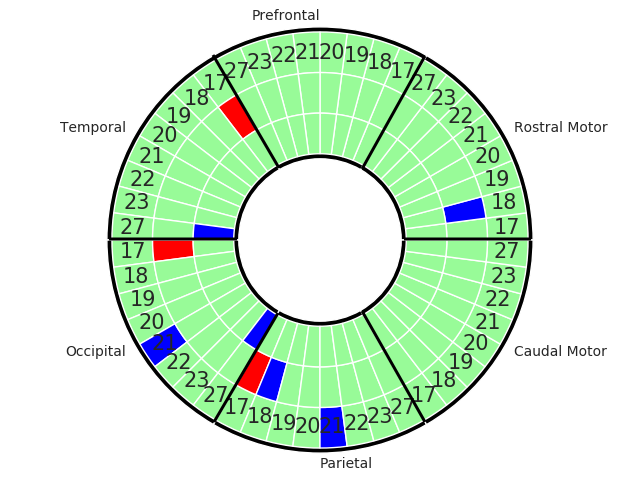


R-CBF inter-ictal (Right / Left)


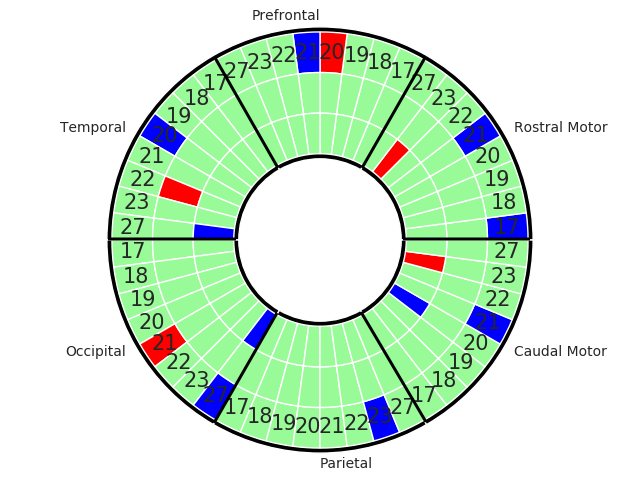

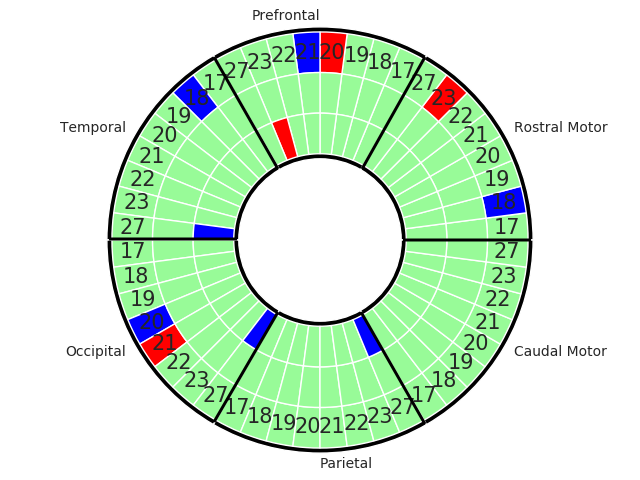


**Supplementary Material Methods**

**Electroencephalography**

Video-EEG recordings lasted 1 to 24 hours and included polygraphic parameters (electrocardiogram (ECG), respiration, surface electromyography (EMG) on both deltoid muscles) using 21 silver chloride cup electrodes placed according to the 10/20 international system using the medial frontal polar as reference electrode. EMG was recorded by two cup electrodes placed 2 cm apart on both deltoid muscles. Signals were amplified (x1000), band-pass filtered at 0.01-97 Hz, and digitized at 256 Hz using the Deltamed Cohérence EEG system (Deltamed/Natus Paris, France). Tracings were retrospectively reviewed by two neurophysiologists (ME, AK). Background activity, inter-ictal anomalies and seizure type(s) were analyzed in all patients. In PDs and ES morphology, topography and frequency of the discharges and eventual associated clinical manifestations were characterized. In patients presenting jerks (myoclonic, clonic, spasms) at ASL-MRI, back averaging was performed on available EEG-EMG files with recorded seizures and locked on the onset of the muscle contraction with an analysis window set to 2 seconds preceding and to 3 seconds following the marked events (Cohérence, Deltamed/Natus, Paris, France).

**MRI and ASL-MRI acquisition**

For both patients and controls, all images were acquired with a standard routine protocol including whole brain T1-weighted and ASL sequences. In children aged six months to five years a sedation protocol (rectal administration of 5 mg/kg pentobarbital) was applied.

Before June 2015, data were acquired on a 1.5 Tesla MRI scanner (Signa HDxt General Electric (GE) Medical System). 3D T1-weighted Fast Spoiled Gradient Echo (FSPGR) images were acquired with the following parameters: 240 axial slices; repetition time (TR) = 16.4 ms; echo time (TE) = 7.2 ms; in-plane resolution = 0.47 x 0.47 mm; slice thickness = 1.2 mm; flip angle (FA) = 13°. ASL perfusion images were performed with a 3D pseudo continuous arterial spin labeling (3D pcASL) sequence using a fast spin echo acquisition with spiral filling of the K space (TR/TE = 4453/10.96 ms; 8 spiral arms x 512 sampling points; Freq = 512; labeling duration (LD) = 1500 ms; post-labeling delay (PLD) = 1025 ms; FA = 155°; matrix size = 128 x 128; in-plane resolution = 1.875 x 1.875 mm; slice thickness = 4 mm; field of view = 24 x 24 cm; bandwidth = 62.50; Imaging Options EDR, Fast, Spiral, Phase 8 Freq, NEX 3.00, Auto Shim On; 40 contiguous axial slices and duration time ≈ 4 minutes.

After June 2015, data were acquired on a Discovery 3.0 Tesla MRI scanner (MR750 General Electric Medical System). 3D T1-weighted FSPGR images were acquired with the following parameters: 156 axial slices; TR = 6.9 ms; TE = 3 ms; in-plane resolution = 1 x 1 mm; slice thickness = 1 mm; FA = 12°. For most deaf patients, Cube T1 images were acquired with these parameters: 374 sagittal slices; TR = 500 ms; TE = 11.2 ms; in-plane resolution = 0.47 x 0.47 mm; slice thickness = 1 mm; FA = 90°. ASL perfusion images were obtained using the same parameters as on the 1.5T system.

**Supplementary Material 1**

*Thalamic network and thalamic neurons properties*

Thalamus is subdivided in relay and association nuclei, the first receiving specific input (sensory, motor, premotor, limbic) and projecting to specific functional areas of cerebral cortex while association nuclei receive and project to association areas (i.e parietal-temporal-occipital/pulvinar; prefrontal/dorsomedial nucleus). The thalamus receives afferents from cortex, cerebellum and GABAergic input from basal ganglia (GPi and SNr). Thalamic projection neurons have two physiological states: tonic and bursting modes depending on the vigilance state. Neuronal recordings in the motor thalamus in awake animals display a wide range of activity from low to high frequencies (1–80 Hz), with brief modulations in activity in relation to movements.^1,2,3,4^ Neurons in the tonic mode can transmit to the cortex information reaching them from specific inputs using trains of action potentials whose frequency is a function of input magnitude. Projection neurons hyperpolarized beyond the tonic range enter a burst mode. During slow wave activity in EEGs or during anesthesia, the activity is organized in bursts that are repeated with consistent periodicity. The firing in the thalamic neurons is characterized by large amplitude slow oscillations in membrane potential with bursts of action potentials during active states called low threshold calcium spikes (LTS). When the membrane is depolarized following a prolonged period of hyperpolarization, the T-type channel is activated (opens) brieﬂy and the inﬂux of calcium ions (IT calcium current) further depolarizes the membrane leading to activation of voltage-gated sodium channels underlying the generation of action potentials.^5^ These LTS bursts occur during slow wave sleep or during drowsiness and have not been reported in awake.

**Supplementary Material 2**

*Basal ganglia networks and medium spiny neurons (MSN) properties*

The principal circuits of the basal ganglia (BG) consist in loops projecting from cerebral cortex to the striatum then returning via the globus pallidus and thalamus back to cortical areas, with precise topographic projections on each level. The putamen is centrally involved in most of the motor functions, the caudate nucleus receives mostly projections from associative areas while the ventral striatum gets inputs from limbic cortex, hippocampus and amygdala.^6^ Thus, the BG are constantly informed about the numerous cortical activities. Individual neurons in the putamen fire in conjunction with particular movements or positions, and stimulation of small areas of the putamen causes movements like contralateral head turning, circling or contraversive limb movements.^7^ Somatosensory areas project primarily to the putamen, the sensorimotor portion of the striatum, terminating on two distinct populations of striatal medium spiny projection neurons (MSNs), which, in turn, send projections either to the external segment of the globus pallidus (GPe), or to the internal segment of the globus pallidus (GPi) and the substantia nigra pars reticulata (SNr), the two output nuclei of the BG. The monosynaptic striatal projection to GPi/SNr is called the direct pathway, while the projection linking the striatum to the GPi/SNr by way of GPe and the subthalamic nucleus is called the indirect pathway.^2^ Axons leaving the striatum, GP and SNr use GABA as neurotransmitter and make inhibitory synapses on their targets. Pallidal and nigral (SNr) neurons provide the final output from the BG and they are tonically active, inhibiting parts of the thalamus.^6,9^ Activation of direct pathway striatal neurons facilitates motor output, whereas activation of indirect pathway neurons inhibits motor output. ^7^ The different ways of motor thalamus control by basal ganglia were conceptualized by Bosch-Bouju and colleagues^5^ proposing three main mechanisms: the rebound model focusing on the ability of basal ganglia to inhibit the thalamus, thus triggering low-threshold-calcium-spike (LTS) bursts, the gating model activating the thalamus through disinhibition (via the direct pathway inhibiting basal ganglia output nuclei which under basal conditions inhibit thalamus), and the entrainment model focusing on the timing of the basal ganglia inputs.

Striatal MSNs are involved in oscillatory multiplexing of neuronal populations and code for interval timing and working memory based on SBF model.^10,11,12,13^ MSNs in the striatum receive a great amount of convergent, multi-modal input from the cortex and exhibit appropriate characteristics to serve as a large-scale coincidence-detector system. The coincident excitatory input from the cortex can drive MSNs into the “UP-state”.^12,14,15^ MSNs within the dorsal striatum act as coincidence detectors of neuronal oscillation patterns (1–15 Hz) from dispersed cortical areas. Phasic bursts from dopaminergic afferents from the ventral tegmental area and the substantia nigra pars compacta synchronize cortical oscillations and reset MSNs, respectively. MSNs integrate and convey EEG activity from the cerebral cortex to the output nuclei of the BG. In vivo, in an anesthetic-free rat, it has been shown that intracellular activity of MSNs depends on vigilance state; during slow-wave sleep, as during anesthesia, MSNs display rhythmic up-down activities correlated with cortical field potentials, while in wakefulness there was a different pattern with disorganized depolarizing synaptic events.^18^ Beside the motor activity, the synchronization of the cortico-striatal neural oscillations occurs during cognitive tasks such as timing process (comparison of sub- and supra-second signal durations) as demonstrated in animal model ^17^ and can be induced by electric stimulations or observed during epileptic seizures as demonstrated in animal model and in patients with epilepsy respectively.^15,19^

**References Supplementary Material**

1. Anderson ME, Turner RS. Activity of neurons in cerebellar-receiving and pallidal-receiving areas of the thalamus of the behaving monkey. *J Neurophysiol*. 1991;66(3):879-893. doi:10.1152/jn.1991.66.3.879

2. Forlano LM, Horne MK, Butler EG, Finkelstein D. Neural activity in the monkey anterior ventrolateral thalamus during trained, ballistic movements. *J Neurophysiol*. 1993;70(6):2276-2288. doi:10.1152/jn.1993.70.6.2276

3. Macia F, Escola L, Guehl D, Michelet T, Bioulac B, Burbaud P. Neuronal activity in the monkey motor thalamus during bicuculline-induced dystonia. *Eur J Neurosci*. 2002;15(8):1353-1362. doi:10.1046/j.1460-9568.2002.01964.x

4. Pessiglione M, Guehl D, Rolland AS, et al. Thalamic neuronal activity in dopamine-depleted primates: evidence for a loss of functional segregation within basal ganglia circuits. *J Neurosci Off J Soc Neurosci*. 2005;25(6):1523-1531. doi:10.1523/JNEUROSCI.4056-04.2005

5. Bosch-Bouju C, Hyland BI, Parr-Brownlie LC. Motor thalamus integration of cortical, cerebellar and basal ganglia information: implications for normal and parkinsonian conditions. *Front Comput Neurosci*. 2013;7. doi:10.3389/fncom.2013.00163

6. Chevalier G, Deniau JM. Disinhibition as a basic process in the expression of striatal functions. 1990;13(7):4.

7. Kravitz AV, Kreitzer AC. Striatal mechanisms underlying movement, reinforcement, and punishment. *Physiol Bethesda Md*. 2012;27(3):167-177. doi:10.1152/physiol.00004.2012

8. Wichmann T, Bergman H, DeLong MR. Basal Ganglia, Movement Disorders and Deep Brain Stimulation: Advances Made Through Non-Human Primate Research. *J Neural Transm*. Published online 2019:19.

9. Shipp S. The functional logic of corticostriatal connections. *Brain Struct Funct*. 2017;222(2):669-706. doi:10.1007/s00429-016-1250-9

10. Matell MS, Meck WH. Cortico-striatal circuits and interval timing: coincidence detection of oscillatory processes. *Brain Res Cogn Brain Res*. 2004;21(2):139-170. doi:10.1016/j.cogbrainres.2004.06.012

11. Fino E, Paille V, Cui Y, Morera-Herreras T, Deniau JM, Venance L. Distinct coincidence detectors govern the corticostriatal spike timing-dependent plasticity. *J Physiol*. 2010;588(Pt 16):3045-3062. doi:10.1113/jphysiol.2010.188466

12. Gu BM, van Rijn H, Meck WH. Oscillatory multiplexing of neural population codes for interval timing and working memory. *Neurosci Biobehav Rev*. 2015;48:160-185. doi:10.1016/j.neubiorev.2014.10.008

13. Kotz, Braun, Schwartze. Cortico-striatal circuits and the timing of action and perception. Published online 2016.

14. Plotkin JL, Day M, Surmeier DJ. Synaptically driven state transitions in distal dendrites of striatal spiny neurons. *Nat Neurosci*. 2011;14(7):881-888. doi:10.1038/nn.2848

15. O’Donnell P, Grace A. Synaptic interactions among excitatory afferents to nucleus accumbens neurons: hippocampal gating of prefrontal cortical input. *J Neurosci*. 1995;15(5):3622-3639. doi:10.1523/JNEUROSCI.15-05-03622.1995

16. Petter EA, Lusk NA, Hesslow G, Meck WH. Interactive roles of the cerebellum and striatum in sub-second and supra-second timing: Support for an initiation, continuation, adjustment, and termination (ICAT) model of temporal processing. *Neurosci Biobehav Rev*. 2016;71:739-755. doi:10.1016/j.neubiorev.2016.10.015

17. Gu BM, Kukreja K, Meck WH. Oscillation patterns of local field potentials in the dorsal striatum and sensorimotor cortex during the encoding, maintenance, and decision stages for the ordinal comparison of sub- and supra-second signal durations. *Neurobiol Learn Mem*. 2018;153:79-91. doi:10.1016/j.nlm.2018.05.003

18. Mahon S, Vautrelle N, Pezard L, et al. Distinct Patterns of Striatal Medium Spiny Neuron Activity during the Natural Sleep-Wake Cycle. *J Neurosci*. 2006;26(48):12587-12595. doi:10.1523/JNEUROSCI.3987-06.2006

19. Aupy J, Wendling F, Taylor K, Bulacio J, Gonzalez-Martinez J, Chauvel P. Cortico-striatal synchronization in human focal seizures. *Brain*. 2019;142(5):1282-1295. doi:10.1093/brain/awz062
